# Two aquaporins, LcPIP1;4 and LcPIP1;4a, cooperatively regulate the onset of dormancy of the terminal buds in evergreen perennial litchi (*Litchi chinensis* Sonn*.*)

**DOI:** 10.1093/hr/uhaf122

**Published:** 2025-05-07

**Authors:** Xue Tian, Zhi-Qun Zhong, Yu Qi, Meng-Meng Ma, Ming-Chao Yang, Dong-Cheng Li, Fang-Yi Zhang, Hui-Cong Wang, Ji-Yuan Shen, Ren-Fang Zeng, Xu-Ming Huang

**Affiliations:** Guangdong Litchi Engineering Research Center, College of Horticulture, South China Agricultural University, 483 Wushan Road, Tianhe District, Guangzhou 510642, China; Guangdong Litchi Engineering Research Center, College of Horticulture, South China Agricultural University, 483 Wushan Road, Tianhe District, Guangzhou 510642, China; Guangdong Litchi Engineering Research Center, College of Horticulture, South China Agricultural University, 483 Wushan Road, Tianhe District, Guangzhou 510642, China; Guangdong Litchi Engineering Research Center, College of Horticulture, South China Agricultural University, 483 Wushan Road, Tianhe District, Guangzhou 510642, China; Key Laboratory of Genetic Resources Evaluation and Utilization of Tropical Fruits and Vegetables (Co-construction by Ministry and Province), Ministry of Agriculture and Rural Affairs, 14 Xingdan Road, Qiongshan District, Haikou 571100, China; Key Laboratory of Tropical Fruit Tree Biology of Hainan Province, Institute of Tropical Fruit Tree, Hainan Academy of Agricultural Sciences, 14 Xingdan Road, Qiongshan District, Haikou 571100, China; Guangdong Litchi Engineering Research Center, College of Horticulture, South China Agricultural University, 483 Wushan Road, Tianhe District, Guangzhou 510642, China; Guangdong Litchi Engineering Research Center, College of Horticulture, South China Agricultural University, 483 Wushan Road, Tianhe District, Guangzhou 510642, China; Guangdong Litchi Engineering Research Center, College of Horticulture, South China Agricultural University, 483 Wushan Road, Tianhe District, Guangzhou 510642, China; Guangdong Litchi Engineering Research Center, College of Horticulture, South China Agricultural University, 483 Wushan Road, Tianhe District, Guangzhou 510642, China; Guangdong Litchi Engineering Research Center, College of Horticulture, South China Agricultural University, 483 Wushan Road, Tianhe District, Guangzhou 510642, China; Guangdong Litchi Engineering Research Center, College of Horticulture, South China Agricultural University, 483 Wushan Road, Tianhe District, Guangzhou 510642, China

## Abstract

Although extensively studied in various plants, the roles of aquaporin proteins in litchi remain unclear. In this study, low moisture content was observed in the dormant terminal buds of litchi. Transcriptome analysis revealed that two aquaporin genes, *PLASMA MEMBRANE INTRINSIC PROTEIN 1;4* (*LcPIP1;4*) and *LcPIP1;5*, could be remarkably inhibited by exogenous ethylene (ETH), which also reduced the moisture content of litchi buds. Quantitative real-time polymerase chain reaction assays indicated that *LcPIP1;4* expression was relatively elevated in the dormancy stage of litchi terminal buds. Inhibition of *LcPIP1;4* in the buds of litchi during the growth stage delayed the onset of dormancy, resulting in a significantly reduced dormancy rate and increased moisture content. Further study indicated that LcPIP1;4 interacts with LcPIP1;4a, and they are capable of self-interaction*.* Silencing of *LcPIP1;4a* in litchi buds resulted in a phenotype consistent with silencing of *LcPIP1;4.* Additionally, simultaneous silencing of both *LcPIP1;4* and *LcPIP1;4a* resulted in a more severe bud dormancy phenotype. Moreover, *LcPIP1;4* was directly upregulated by LcRAP2.4. Silencing of *LcRAP2.4* also delayed the onset of dormancy in litchi terminal buds, which is regulated by LcSVP2. ETH treatment at 1000 mg/l significantly downregulated the expression of *LcPIP1;4* and *LcRAP2.4,* but had no significant effect on *LcPIP1;4a*. In contrast, abscisic acid (ABA) treatment at 200 mg/l significantly upregulated the expression of *LcPIP1;4*, *LcPIP1;4a*, and *LcRAP2.4*. Combined treatment with ETH and ABA exerted a stronger inhibitory effect on the bud break and upregulated *LcPIP1;4* and *LcRAP2.4* to lower degrees than ABA alone, suggesting that ABA reversed the inhibitory effect of ETH on the expression of *LcPIP1;4* and *LcRAP2.4*. ABA treatment and combined treatment with ETH and ABA effectively reduced the moisture content of the terminal buds. These results demonstrate that LcRAP2.4, LcPIP1;4, and LcPIP1;4a play a vital role in dormancy onset of litchi terminal buds by regulating moisture levels.

## Introduction

Plants exhibit unique growth patterns, reflecting an evolutionary adaptation to external conditions and the distribution of nutrients among various tissues [1, 2]. Temperate deciduous woody plants undergo an annual cycle of bud outgrowth in spring, active growth in summer, growth cessation in autumn, and bud dormancy in winter. The photoperiod is a key environmental cue to determine the optimal timing of trees to cease seasonal growth [3]. The seasonal growth and dormancy patterns of deciduous fruit trees serve as an important strategy to survive harsh environmental conditions. Unlike deciduous temperate trees, many evergreen fruit species from subtropical and tropical regions, such as litchi, citrus, and mango, undergo a recurrent growth pattern, featuring several flush growth cycles throughout the year [4–6]. Nonenvironmentally induced bud dormancy occurring between flush growths temporally terminates the food supply of pests that feed on tender flushes, serving as an efficient way to prevent damage caused by pest outbreaks [7].

The processes that govern the onset and release of bud dormancy present fascinating subjects for research. While there has been extensive research and a deep understanding of the regulation of bud dormancy of temperate tree species, this process in tropical evergreens remains unclear. Nevertheless, the insights gained from temperate tree species can serve as a valuable reference to explore developmental dormancy of tropical evergreens. In temperate trees, light (especially the photoperiod), temperature, and water availability are essential to initiate, sustain, and terminate dormancy [8]. Physiological factors, such as hormones, sugars, polyamines, and enzymes, also play a role in dormancy regulation [8]. The MADS-box family of transcription factors (TFs), SHORT VEGETATIVE PHASE (SVP) or Dormancy-Associated MADS-box (DAM) proteins, reportedly play significant roles in bud dormancy regulation [4, 9–11]. In addition, the roles of plant hormones in bud dormancy regulation of perennial trees have been extensively studied [12, 13]. In pear trees, abscisic acid (ABA), gibberellins, brassinosteroid, and jasmonic acid have been identified as key regulators of bud dormancy [14]. Ethylene (ETH) has been recognized for its significant functions in modulating bud dormancy and flowering in temperate fruit trees [15]. Furthermore, transcriptome analysis indicated that most auxin-related genes are downregulated during dormancy in the Japanese apricot [16], indicating that auxin also participates in dormancy regulation of perennial fruit trees. A previous study demonstrated that ABA and ETH enforce dormancy of the terminal buds of the tropical evergreen fruit tree litchi by upregulating *LcSVP2* [4].

The entry and exit of bud dormancy in temperate trees also involve changes to the water content of the bud, which decreases during the entry of dormancy and increases after dormancy release before bud break [17–19]. A significant reduction in the uptake of ^2^H-labeled water by buds was noted during the entry of dormancy, and there was a direct relationship between the moisture content of the buds and the thermal time leading to bud break [20]. In the tropical evergreen litchi, the terminal bud shrinks, hardens, and becomes dark during the entry of dormancy, then swells and turns green during the bud break stage [7], indicating similar changes to the water content during bud onset and release in tropical plants.

Several studies indicate that aquaporins proteins (AQPs), which are responsible for transmembrane water movement, play a crucial role in the regulation of plant dormancy [21–23]. AQPs, commonly referred to as major intrinsic proteins (MIPs), play a crucial role in the regulation of water transport in various higher plant species. Based on their subcellular localization and sequence similarity, AQPs of plants are divided into five subfamilies: plasma membrane intrinsic proteins (PIPs), tonoplast intrinsic proteins (TIPs), NOD26-like intrinsic proteins (NIPs), small basic intrinsic proteins (SIPs), and the recently identified X intrinsic proteins (XIPs), respectively [24]. The PIP subfamily is further subdivided into the PIP1 and PIP2 groups.

Owing to the crucial roles in numerous physiological processes, functional regulation of AQPs in plants has been a significant area of research over the past 10 years. The expression levels of *AQPs* have been linked to plant growth, which involves cell division, expansion, and differentiation, as well as bud dormancy and flowering [19, 23, 25, 26]. Overexpression of *PIP1b* has been reported to significantly modify the vegetative and reproductive growth phases of *Arabidopsis*, and the transgenic plants demonstrate enhanced growth, greater transpiration rates, with elevated stomatal density, and improved photosynthetic efficiency [27]. *AQP*s have been identified in the genomes of several plants, including *Brassica oleracea* [28], *Vitis vinifera* [29], wheat [30], cotton [31], sugar beet [32], and tomato [33], among others, highlighting their diverse functions across numerous species.

Litchi is a significant tropical or subtropical fruit with substantial economic value. Since there has been limited research, the functions of AQPs in litchi remain largely unknown. Therefore, the aim of the present study was to clarify the mechanisms employed by the AQPs LcPIP1;4 and LcPIP1;4a to regulate the dormancy of litchi terminal buds. The results of this study offer new insights into the regulation of bud dormancy in tropical evergreen fruit trees.

## Results

### Low moisture content in litchi terminal dormancy buds

**Figure 1:**
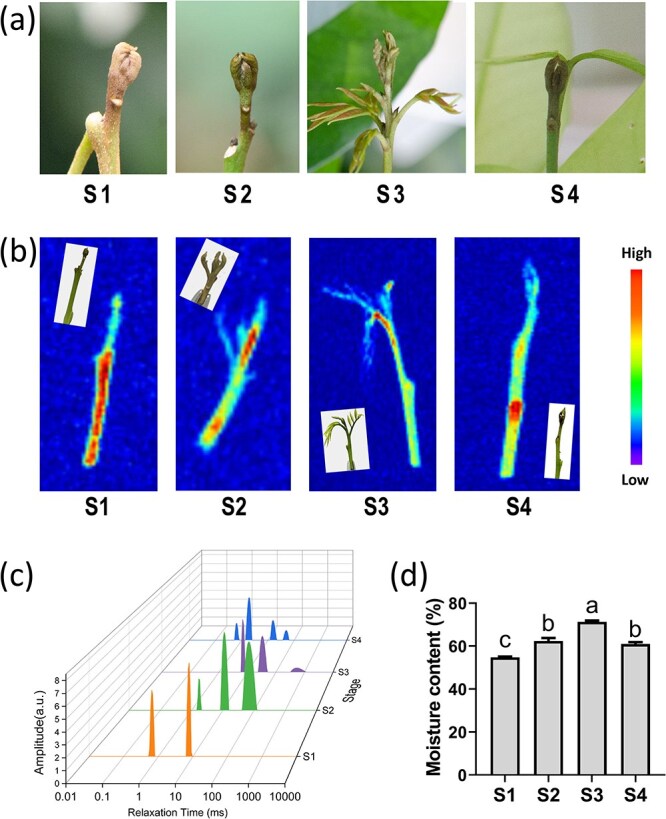
Analysis of the moisture content of litchi terminal buds at various developmental stages. (a) Litchi terminal buds at different developmental stages (S1 represents dormancy stage, S2 represents bud break stage, S3 represents rapid growth stage, and S4 represents growth arrest stage). (b) NMR pseudo-color maps of shoot tips at different stages. (c) T2 relaxation spectra of litchi buds at different stages. (d) Moisture content of the terminal buds at various stages.

When entering the dormancy stage, litchi terminal buds display a dry appearance (Fig. 1a). Low-field nuclear magnetic resonance (LF-NMR) and magnetic resonance imaging (MRI) techniques were employed to detect the moisture content of buds at various developmental stages (Fig. 1b). The MRI results clearly indicate abundant moisture in the shoot stems at S1, whereas a very weak moisture signal was present in the terminal buds (Fig. 1b), indicating poor diffusion of water from the stem to the terminal bud. At S2, abundant moisture was observed in the rudimentary leaves and terminal tender stems, with a moisture signal relatively similar between the stem and the opening terminal bud, suggesting smooth diffusion of water from the shoot stems to the distal bud (Fig. 1b). Similar to S2, the moisture signal was strongest in the growing leaves at S3 (Fig. 1b). At S4, water activity in the terminal bud was notably decreased, but increased in the stem, indicating reduced moisture diffusion from the stem to the bud, marking reentry of the terminal bud into the dormancy stage (Fig. 1b).

The transverse relaxation time (T2), which indicates the moisture status in the bud, is highly correlated with water mobility. The water phase at T2 ranging from 0 to 10 ms is considered bound water (T21), 10 to 100 ms (T22) as semibound water, and 100 to 1000 ms (T23) as free water. During S1, only two T21 water phase peaks were detected, indicating that the terminal buds in the dormancy stage contained only bound water (Fig. 1c). At S2, T2 was increased as compared with S1, with two T22 water phase peaks, indicating a transition from bound water to free water, leading to an increase in the quantity of semibound water in the terminal bud during bud beak (Fig. 1c). At S3, one peak was detected in T21, T22, and T23, and the T23 peak occurred at close to 1000 ms, indicating that the growing bud contained highly free water (Fig. 1c). During S4, the water phase in T23 disappeared and two peaks each in T21 and T22 were detected, indicating that the free water content was reduced and both the semibound and bound water contents increased as the terminal buds reentered the dormancy stage (Fig. 1c).

The moisture content of the terminal bud was the lowest at S1, increased during the bud break stage (S2), became the highest during flush growth at S3, and decreased during growth cessation at S4 (Fig. 1d). These results indicate that the water status of the terminal buds varied across different stages, and that water mobility is closely associated with dormancy onset and release, which compelled exploration of the roles of AQPs in this dynamic of the terminal bud. To investigate the regulatory functions of AQPs in the dormancy of litchi terminal buds, a total of 30 *AQP* genes were identified in litchi, and their expression was examined at four distinct developmental stages of the terminal buds. Except for three genes (LITCHI030529, LITCHI030532, and LITCHI011893), for which expression was not detected, all other genes were found to be expressed in the litchi terminal buds (Fig. S1). The results revealed that seven *AQP* genes (LITCHI019534, LITCHI027788, LITCHI024831, LITCHI009087, LITCHI002040, LITCHI019993, and LITCHI008500) exhibited high expression levels in S2 during the bud break stage and low expression levels in S4 during the growth arrest stage or at the onset of dormancy (Fig. S1). Conversely, the expression levels of eight *AQP* genes (LITCHI020024, LITCHI001953, LITCHI025727, LITCHI025728, LITCHI027284, LITCHI027537, LITCHI018243, and LITCHI008267) were relatively low in S2 but high in S4 (Fig. S1). These findings suggest that these 15 *AQP* genes may be involved in dormancy regulation of litchi terminal buds.

### *LcPIP1;4* is inhibited by ETH and exhibits high expression during litchi bud dormancy

**Figure 2:**
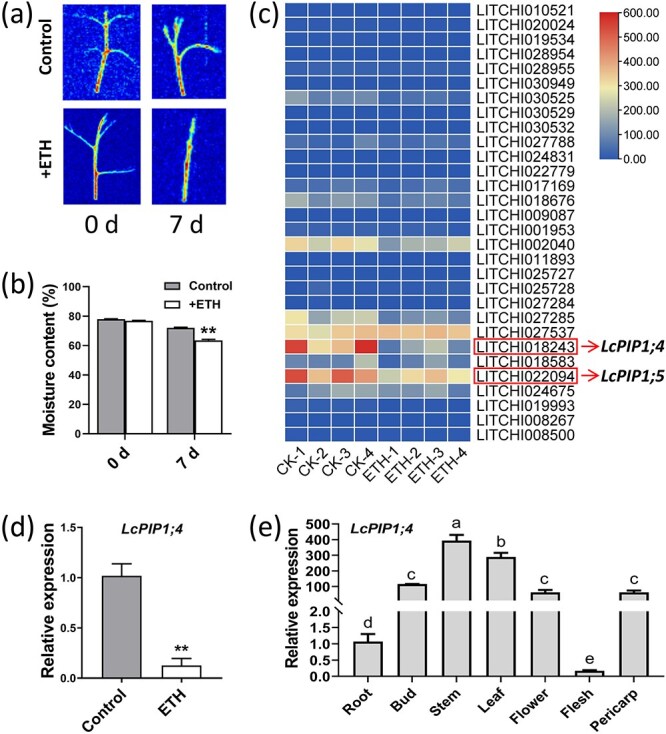
Identification of *LcPIP* genes in response to ETH treatment. (a) NMR pseudo-color maps of terminal buds subjected to ETH treatment as compared to the control group. (b) Moisture content of the terminal buds of ETH-treated versus control samples. (c) Heat map analysis of *LcPIP* gene expression levels in the terminal buds following ETH treatment. (d) Expression levels of *LcPIP1;4* based on qRT-PCR analysis in the control and the ETH-treated bud samples. (e) Expression levels of *LcPIP1;4* in different organ tissues of litchi, including roots, buds, stems, leaves, flowers, flesh, and pericarp of fruit. Error bars indicate the standard error of the mean (*n* = 3). ^**^Indicates a significant difference at *P* < 0.01, as determined by Student’s *t*-test. Different letters above the bars indicate significant difference at *P* < 0.05 among stages or tissues based on LSD multiple range test.

Previously, we found that ETH effectively induced dormancy onset and maintained dormancy of litchi terminal buds [4, 34]. To further investigate the impact of ETH treatment on the moisture content in litchi terminal buds, the moisture content of the terminal buds was measured on post-treatment day 7. The results indicate that ETH treatment considerably reduced the moisture content of the buds (Fig. 2a and b). Consequently, comparative transcriptomic analysis between the ETH-treated and control bud samples was conducted, focusing on the expression levels of AQP-encoding genes. A heat map expression analysis of all identified AQP-encoding genes revealed that the expression levels of LITCHI018243 and LITCHI022094 tended to decrease following ETH treatment (Fig. 2a and Table S1). These two genes were designated as *LcPIP1;4* (LITCHI018243) and *LcPIP1;5* (LITCHI022094) (Fig. 2c). ETH-induced dormancy onset downregulated expression of *LcPIP1,4* and *LcPIP1;5*, suggesting an association of these two genes with the regulatory effect of water on litchi bud dormancy. However, there were no significant differences in *LcPIP1;5* expression across the four developmental stages of the litchi bud (Fig. S1). Therefore, we focused on *LcPIP1;4* and proposed that it might play a crucial role in regulation of bud dormancy. The quantitative real-time polymerase chain reaction (qRT-PCR) results confirmed that the expression of *LcPIP1;4* was significantly downregulated by ETH (Fig. 2d). Further investigations of various tissues of litchi revealed that expression of *LcPIP1;4* was relatively low in the root and flesh of fruit, and high in other tissues (Fig. 2e).

### Inhibition of *LcPIP1;4* expression delayed the dormancy onset of litchi terminal buds

**Figure 3:**
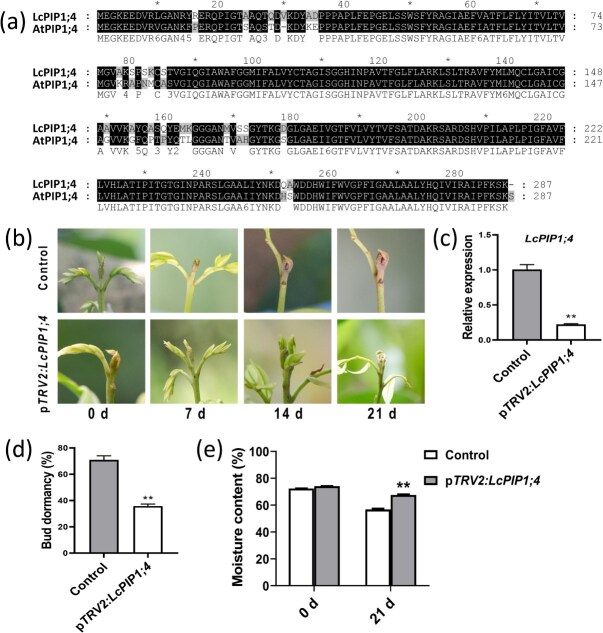
Sequence and functional analyses of LcPIP1;4. (a) Protein sequence alignment of LcPIP1;4 and *Arabidopsis* AtPIP1;4. (b) Effect of *LcPIP1;4* silencing on litchi bud dormancy. (c) Expression levels of *LcPIP1;4* in p*TRV2:LcPIP1;4* and control buds. (d) Dormancy rates of p*TRV2:LcPIP1;4* and control buds. Error bars represent the standard error of the mean. (e) Moisture content in the p*TRV2:LcPIP1;4* and control buds. ^**^Indicates a significant difference at *P* < 0.01, as determined by Student’s *t*-test.

A phylogenetic tree was constructed to investigate the homologous relationship of LcPIP1;4 with the AtPIP family members of *Arabidopsis*. The results indicated that the homology between LcPIP1;4 and AtPIP1;4 is very high (Fig. S2). Sequence alignment revealed high sequence similarity of LcPIP1;4 and the *Arabidopsis* AtPIP1;4 protein (Fig. 3a). To explore the function of LcPIP1;4, a gene silencing experiment was performed. Terminal buds at S3 were selected for silencing of *LcPIP1;4* and growth status was recorded 7, 14, and 21 days post-virus-induced gene silencing (VIGS) injection (Fig. 3b). The treatment resulted in significantly reduced expression of *LcPIP1;4* (Fig. 3c) and delayed onset of bud dormancy (Fig. 3b), with a significantly lower percentage of bud dormancy observed at day 21 (Fig. 3d). In addition, after the silencing of *LcPIP1;4*, the moisture content was significantly elevated in the terminal buds (Fig. 3e). These results suggest that LcPIP1;4 promotes water loss from the terminal buds and serves as a promoter of dormancy onset.

### LcPIP1;4 interacts with LcPIP1;4a

**Figure 4:**
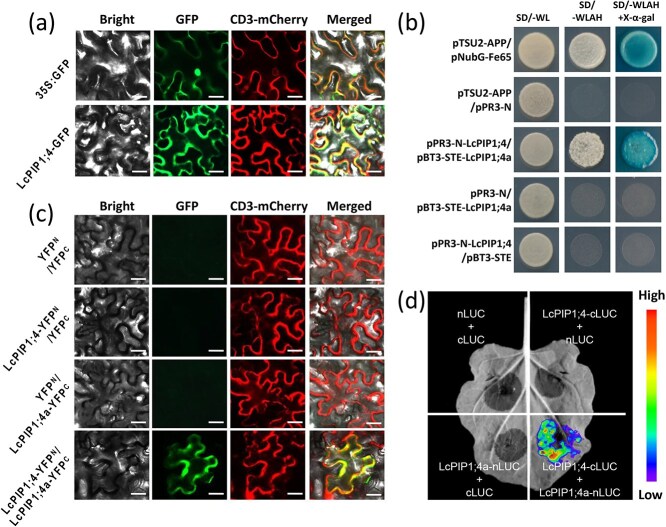
Subcellular localization of LcPIP1;4 and the interaction between LcPIP1;4 and LcPIP1;4a. (a) Subcellular location of LcPIP1;4 in tobacco leaf cells. CD3-mCherry represents the plasma membrane marker. Bars = 50 μm. (b) Split-ubiquitin Y2H assay revealing the interaction between LcPIP1;4 and LcPIP1;4a. pTSU2-APP with pPR3-N, pPR3-N with pBT3-STE-LcPIP1;4a, and pPR3-N-LcPIP1;4 with pBT3-STE were used as negative controls. The experiment was performed independently three times. (c) The BiFC assay in tobacco leaves shows the interaction between LcPIP1;4 and LcPIP1;4a. CD3-mCherry represents the plasma membrane marker. Bars = 50 μm. (d) The LCI assays showed that LcPIP1;4 interacts with LcPIP1;4a. nLUC + cLUC, nLUC + LcPIP1;4-cLUC, and LcPIP1;4a-nLUC + cLUC combinations were used as negative controls.

Subcellular localization studies have shown that LcPIP1;4 is a plasma membrane protein (Fig. 4a). To explore the regulatory pathway involving LcPIP1;4, screening of the Y2H library was conducted using LcPIP1;4 as the bait alongside a prey cDNA library derived from litchi terminal buds. Notably, another PIP protein family member, designated LcPIP1;4a (LITCHI027537), was found to potentially interact with LcPIP1;4. To further validate the interaction between LcPIP1;4 and LcPIP1;4a, we conducted additional Y2H test (Fig. 4b). The recombinant plasmids pBT3-STE-LcPIP1;4 and pPR3-N-LcPIP1;4a were cotransformed into yeast NMY51 cells and cultured on synthetic-defined (SD) medium lacking Leu, Trp, His, and Ade (SD/−Leu/−Trp/−His/−Ade), with the empty vectors pPR3-N and pTSU2-APP serving as negative controls. The Y2H assay results showed that yeast cells coexpressing pBT3-STE-LcPIP1;4 and pPR3-N-LcPIP1;4a thrived on SD/−Leu/−Trp/−His/−Ade. In contrast, the negative control groups, which included transformations of pBT3-STE-LcPIP1;4 with pPR3-N, or pBT3-STE with pPR3-N-LcPIP1;4a, failed to grow, indicating an interaction between LcPIP1;4 and LcPIP1;4a (Fig. 4b).

The bimolecular fluorescence complementation (BiFC) assay was conducted to further confirm the interaction between LcPIP1;4 and LcPIP1;4a. The findings indicated that the nuclei of cells cotransformed with both LcPIP1;4 and LcPIP1;4a exhibited a green fluorescent protein (GFP) signal (Fig. 4c). In contrast, there was no GFP signal detected in the nuclei of cells transformed either with LcPIP1;4 and an empty plasmid or with an empty plasmid and LcPIP1;4a (Fig. 4c). In addition, luciferase complementation imaging (LCI) assays were subsequently performed, in which robust luciferase (LUC) signals were only present in samples cotransformed with *LcPIP1;4-cLUC* and *LcPIP1;4a-nLUC*, while the negative controls showed no LUC signal. These observations support the conclusion that LcPIP1;4 interacts with LcPIP1;4a (Fig. 4d). Numerous studies have shown that PIP proteins can form homodimeric or multimeric complexes to carry out related functions [35–37]. The interactions between LcPIP1;4 and LcPIP1;4a suggest that the formation of PIP complexes also occurs in litchi. Notably, the Y2H and BiFC results further confirmed that both proteins could interact with themselves (Fig. S3).

### LcPIP1;4a is located in the plasma membrane and *LcPIP1;4a* is highly expressed in the litchi terminal buds at dormancy stage

**Figure 5:**
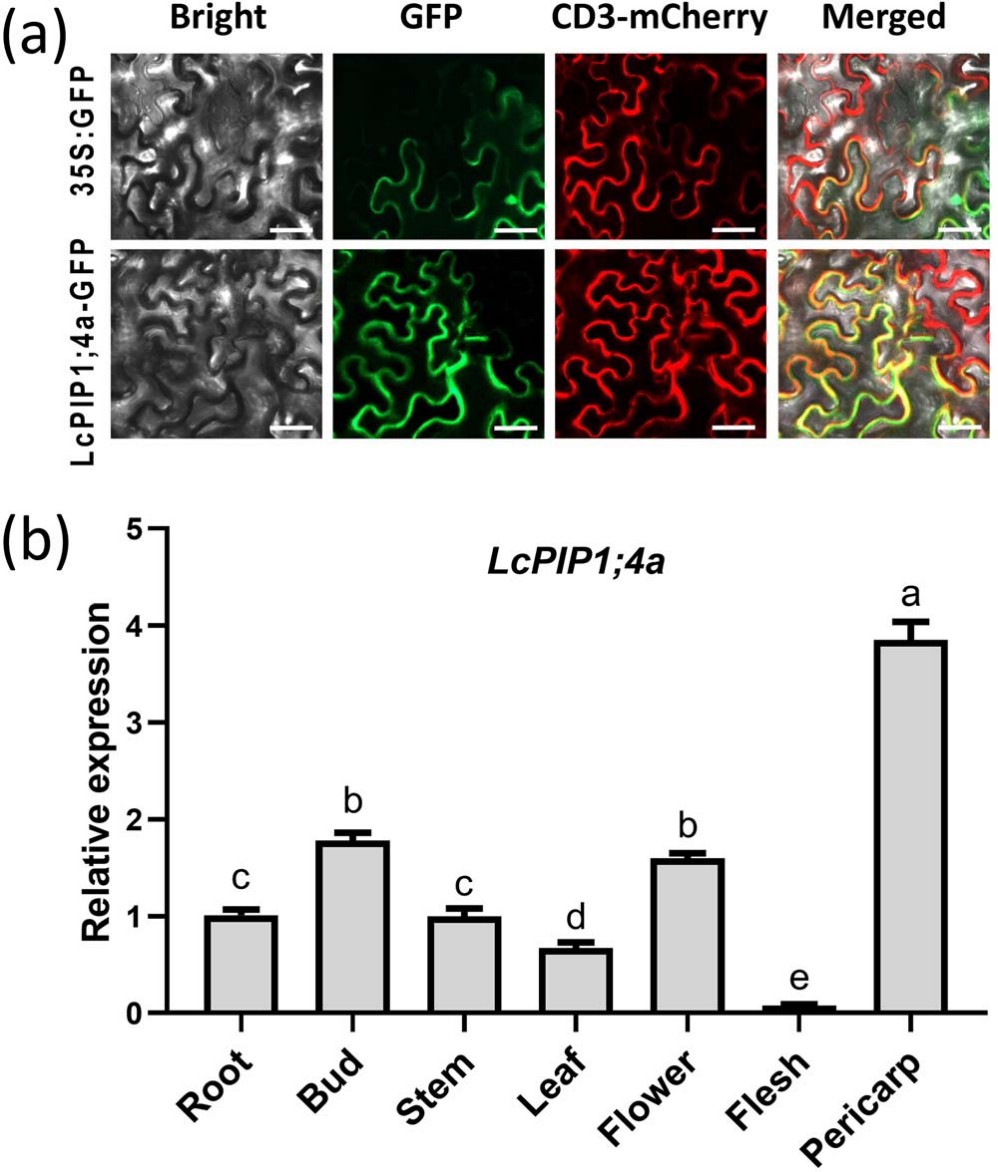
The expression characteristics of *LcPIP1;4a*. (a) Subcellular localization of LcPIP1;4a in tobacco leaf cells. CD3-mCherry represents the plasma membrane marker. Bars = 50 μm. (b) Expression levels of *LcPIP1;4a* in various litchi tissues. Error bars represent the standard error of the mean (*n* = 3). Different letters above the bars indicate significant difference at *P* < 0.05 among stages or tissues based on the LSD multiple range test.

Sequence alignment of LcPIP1;4, LcPIP1;4a, and *Arabidopsis* AtPIP1;4 found a high degree of sequence similarity among these proteins (Fig. S4). Subcellular localization studies indicated that LcPIP1;4a is a plasma membrane protein, consistent with localization of LcPIP1;4 (Fig. 5a). Previous qRT-PCR analysis of *LcPIP1;4a*/LITCHI027537 have shown that the expression of this gene was the highest expression level during dormancy entry (S4), followed by dormancy period (S1) (Fig. S1), suggesting that LcPIP1;4a may play a regulatory role in terminal bud dormancy. Additionally, *LcPIP1;4a* expression was also assessed in different tissues and tissues, showing high levels in flowers, buds, and pericarp (Fig. 5b).

### Silencing of *LcPIP1;4a* delayed the dormancy onset of litchi buds and silencing of both *LcPIP1;4a* and *LcPIP1;4* exacerbated this phenotype

**Figure 6:**
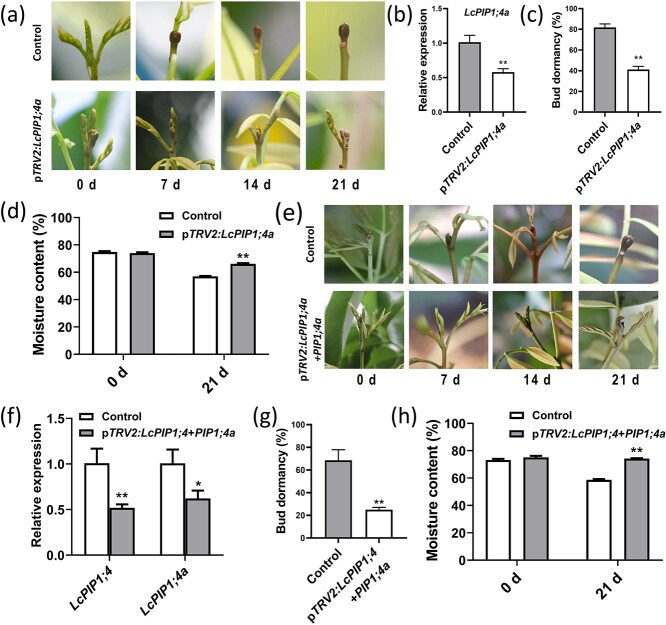
Functional identification of LcPIP1;4a. (a) Silencing of *LcPIP1;4a* expression in litchi buds delayed the onset of dormancy. (b) The expression levels of *LcPIP1;4a* in p*TRV2:LcPIP1;4a* and control buds. (c) Dormancy rates of p*TRV2:LcPIP1;4a* and control buds. (d) Moisture content of the p*TRV2:LcPIP1;4a* and control buds. (e) Phenotype of the terminal buds resulting from simultaneous silencing of *LcPIP1;4* and *LcPIP1;4a*. (f) Expression levels of *LcPIP1;4* and *LcPIP1;4a* in p*TRV2:LcPIP1;4* + p*TRV2:LcPIP1;4a* VIGS and control buds. (g) Dormancy rates of p*TRV2:LcPIP1;4* + p*TRV2:LcPIP1;4a* as compared to control buds. (h) Moisture content of p*TRV2:LcPIP1;4* + p*TRV2:LcPIP1;4a* and control buds. Error bars represent the standard error of the mean. ^**^Indicates a significant difference at *P* < 0.01, as determined by Student’s *t*-test.

VIGS analysis was performed to investigate the regulatory role of LcPIP1;4a in the dormancy of litchi buds. The morphology of terminal buds at 7, 14, and 21 days following VIGS treatment was observed and recorded (Fig. 6a). The results demonstrated that silencing of *LcPIP1;4a* effectively delayed the dormancy onset of litchi buds (Fig. 6a). At 21 days after VIGS treatment, the VIGS group exhibited reduced expression of *LcPIP1;4a* and a considerably lower dormancy rate of terminal buds as compared to the control group (Fig. 6b and c). Also, at 21 days after silencing of *LcPIP1;4a*, the moisture content in the terminal buds was significantly higher than that of the control group (Fig. 6d), suggesting that LcPIP1;4a facilitates water loss in the terminal buds.

In addition, we further investigated the phenotype observed when both *LcPIP1;4a* and *LcPIP1;4* were silenced in the terminal buds (Fig. 6e). The results indicated that simultaneous silencing of *LcPIP1;4a* and *LcPIP1;4* significantly inhibited their expression levels in the terminal buds (Fig. 6f). As compared to the control group, the delayed dormancy phenotype of the terminal buds was more pronounced in the VIGS group (Fig. 6e). After 21 days, most buds treated with VIGS injection did not enter the dormant state, resulting in a bud dormancy rate of only 25% (Fig. 6g). Water content measurements revealed that after silencing of the *LcPIP1;4a* and *LcPIP1;4* genes, the water content in the terminal bud was significantly higher than that of the control (Fig. 6h), suggesting that water loss was inhibited in the VIGS group. These findings indicate that simultaneous silencing of both *LcPIP1;4a* and *LcPIP1;4* exacerbated the delayed dormancy effect in litchi terminal buds.

### LcRAP2.4 directly binds to the *LcPIP1;4* promoter and promotes its expression

**Figure 7:**
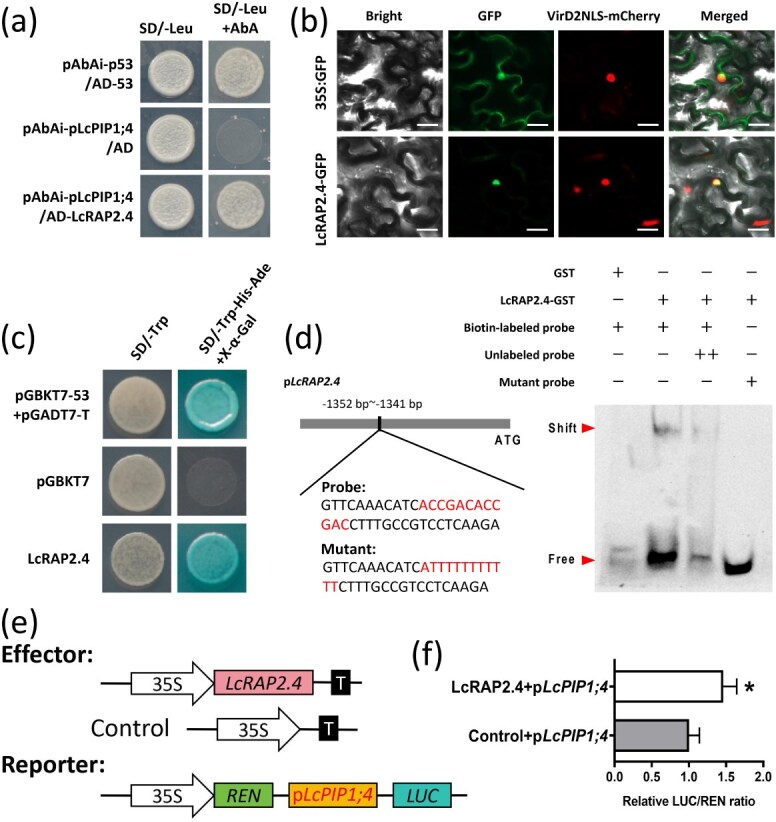
LcRAP2.4 binds to the promoter of *LcPIP1;4* and promotes its expression. (a) The Y1H assay indicates that LcRAP2.4 specifically binds to the *LcPIP1;4* promoter. (b) Subcellular localization of LcRAP2.4 in tobacco leaf cells is shown, with the VirD2NLS-mCherry indicating the presence of nuclear marker. Bars = 50 μm. (c) Analysis of transcriptional activity in yeast demonstrates that LcRAP2.4 possesses transcriptional activation capability. (d) The EMSA confirmed that LcRAP2.4 binds to the DRE/CRT element (ACCGACACCGAC) within the *LcPIP1;4* promoter. (e) A schematic diagram illustrating the composition of the effector and reporter components of the dual-luciferase reporter assays. (f) The dual-luciferase reporter assays revealed that LcRAP2.4 stimulated the expression of *LcPIP1;4*, with the LUC/REN ratio indicating the relative activity of the *LcPIP1;4* promoter. Error bars represent the standard error of the mean (*n* = 3). *Denotes a significant difference at *P* < 0.05, based on Student’s *t*-test.

To investigate the upstream regulatory mechanism of LcPIP1;4, the promoter of *LcPIP1;4* from litchi was cloned and a yeast one-hybrid (Y1H) library was constructed using promoter fragments as bait. As a result, LcRAP2.4 (LITCHI010452) was identified as a potential TF that may bind to the *LcPIP1;4* promoter. Phylogenetic tree analysis confirmed that the *LcRAP2.4* is homologous to the *Arabidopsis* gene that encodes RAP2.4, a member of the DREB subfamily A-6 within the ERF/AP2 TF family (Fig. S5a). Sequence analysis indicated that both proteins possessed conserved ERF/AP2 domains (Fig. S5b). The Y1H assay was conducted to confirm that LcRAP2.4 bound to the *LcPIP1;4* promoter. The results showed that in the Leu-deficient culture medium (−Leu) supplemented with AbA, the positive control yeast and the cotransformed yeast with pAbAi-pLcPIP1;4 and AD-LcRAP2.4 grew normally, while the negative control group did not, indicating that LcRAP2.4 can bind to the *LcPIP1;4* promoter (Fig. 7a).

The subcellular localization results revealed that the LcRAP2.4 is located in the nucleus (Fig. 7b). Subsequently, the transcriptional activity of LcRAP2.4 was analyzed using yeast, which confirmed function as a transcriptional activator (Fig. 7c). Previous studies have demonstrated that genes in the ERF TF family can bind to DRE and GCC-box elements [38, 39]. Sequence analysis of the promoter of *LcPIP1;4* revealed a binding site of DRE (ACCGACACCGAC) for LcRAP2.4 located −1352 to −1341 bp upstream of the start codon (Fig. 7d). The electrophoretic mobility shift assay (EMSA) also confirmed that LcRAP2.4 could bind to the sequence of DRE element (Fig. 7d). The dual luciferase assay indicated that the LUC/REN ratio in the experimental group of tobacco leaves cotransformed with LcRAP2.4 and LcPIP1;4 was markedly higher as compared to the control group, suggesting that LcRAP2.4 TF promotes the transcription of *LcPIP1;4* (Fig. 7e and f).

### Silencing of *LcRAP2.4* in litchi terminal buds delayed the onset of dormancy and reduced expression of *LcPIP1;4*

**Figure 8:**
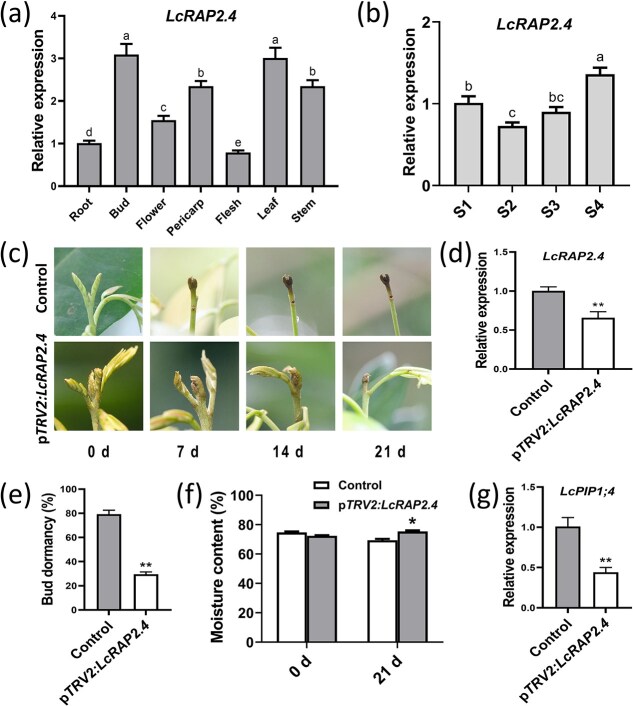
Functional analysis of LcRAP2.4. (a) Expression of *LcRAP2.4* in different tissues. (b) Expression level of *LcRAP2.4* in terminal buds at different stages. (c) Phenotypic changes resulting from the inhibition of *LcRAP2.4* expression in litchi buds. (d) The expression levels of *LcPIP1;4a* in p*TRV2:LcPIP1;4* and control buds. (e) Dormancy rates of p*TRV2:LcPIP1;4a* and control buds. (f) Moisture content of p*TRV2:LcRAP2.4* and control buds. (g) Expression levels of *LcPIP1;4* in p*TRV2:LcRAP2.4* and control buds. Different letters above the bars indicate significant difference at *P* < 0.05 among stages or tissues based on the LSD multiple range test.

The expression level of *LcRAP2.4* was highest in the terminal buds and leaves (Fig. 8a). In the terminal buds, the expression of *LcRAP2.4* was highly expressed during the dormancy stage (S1), decreased during the bud break stage (S2) and rapid growth stage (S3), and subsequently increased during the growth cessation stage (S4) (Fig. 8b). These results indicate that LcRAP2.4 may play an important role in dormancy of litchi terminal buds.

A VIGS assay was employed to reduce the expression of *LcRAP2.4* in the terminal buds at S3 (Fig. 8c). Notably, at 21 days post-VIGS treatment, the reentry into dormancy of the terminal buds was postponed, and there was a significant decrease in *LcRAP2.4* expression (Fig. 8d), the average dormancy rate of the buds also significantly declined (Fig. 8e), and the rate of water loss in the terminal bud following *LcRAP2.4* gene silencing was lower than that of the control (Fig. 8f). These findings imply that, akin to LcPIP1;4 and LcPIP1;4a, LcRAP2.4 likely serves as a crucial regulator of dormancy onset of litchi terminal buds. Furthermore, the expression levels of *LcPIP1;4* was notably reduced in the p*TRV2:LcRAP2.4* buds (Fig. 8g). This indicates that LcRAP2.4 enhances the expression of *LcPIP1;4*. However, the qRT-PCR results indicated that LcRAP2.4 did not promote the expression of *LcPIP1;4a* (Fig. S6a), and the Y1H assays also demonstrated that LcRAP2.4 could not bind to the promoter of *LcPIP1;4a* (Fig. S6b). Additionally, analysis of the *LcPIP1;4* and *LcPIP1;4a* promoter sequences identified four DRE elements in the *LcPIP1;4* promoter and only one DRE element in the *LcPIP1;4a* promoter (Table S2).

### ETH and ABA influenced expression of *LcRAP2.4* and *LcPIP1;4*

**Figure 9:**
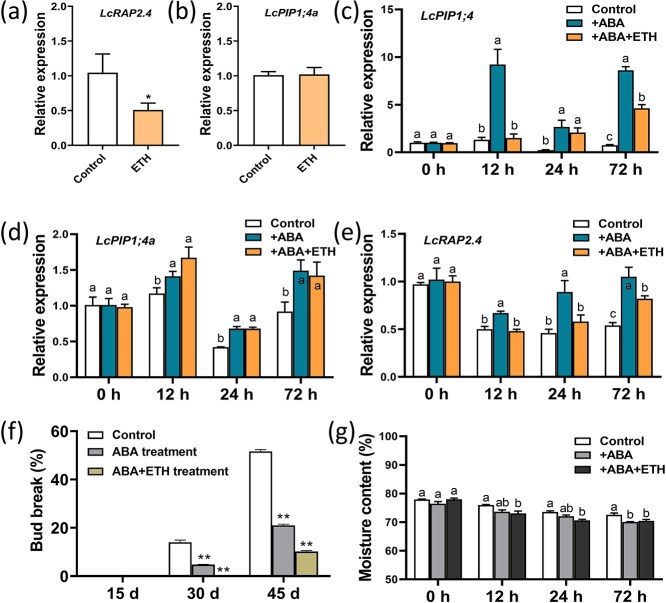
ABA and ETH have opposite effects on expression of the *LcPIP1;4*, *LcPIP1;4a*, and *LcRAP2.4* genes. (a) Effects of ETH treatment on the expression of *LcRAP2.4* and (b) *LcPIP1;4a* in the terminal buds. (c) Effects of ABA and ABA + ETH treatment on expression of *LcPIP1;4*, (d) *LcPIP1;4a*, and (e) *LcRAP2.4* in the terminal buds. (f) Effect of exogenous ABA and ET treatment on bud break. (g) Moisture content of the terminal buds following treatment with ABA and ABA + ETH. ^**^Indicates a significant difference at *P* < 0.01, as determined by Student’s *t*-test. Different letters above the bars indicate significant difference at *P* < 0.05 based on the LSD multiple range test.

ETH is a crucial regulator of the dormancy of litchi buds [4, 34, 40]. The transcriptome and qRT-PCR results previously mentioned indicate that *LcPIP1;4* is inhibited by ETH. ETH also inhibited the expression of *LcRAP2.4* but had no significant effect on *LcPIP1;4a* (Fig. 9a and b). Since ABA plays a significant role in dormancy entry and maintenance, the effects of ABA and the combination of ABA and ETH on the expression of *LcRAP2.4*, *LcPIP1;4*, and *LcPIP1;4a* were examined (Fig. 9c–e). Notably, treatment with ABA alone resulted in a significant increase in their expression from 12 h after treatment. However, combined treatment with ABA and ETH had significantly increased the expression levels of all three genes from 24 h after treatment, and the effect was less significant than ABA treatment alone (Fig. 9c–e). These results suggested that ABA weakens the inhibitory effect of ETH on *LcRAP2.4* and *LcPIP1;4*. The bud break rate was significantly reduced by ABA treatment as compared to the control, and the combination of ABA and ETH enhanced suppression of bud break (Fig. 9f).

In addition, the moisture content of the litchi terminal buds treated with hormones was assessed. The results indicate that the moisture content of buds treated with ABA alone or in combination with ETH was lower than that of the untreated control (Fig. 9g). Within 24 h after treatment with ABA alone, there was no significant difference in the moisture content of the buds as compared to the control. However, following simultaneous treatment with ABA and ETH, the moisture content was significantly lower in the buds than that of the control (Fig. 9g). After 72 h, regardless of whether ABA was applied alone or in conjunction with ETH, the moisture content of the buds remained significantly lower than that of the control (Fig. 9g). These findings suggest that ABA and ETH promote water loss in the litchi terminal buds, with simultaneous application yielding more pronounced effects.

### Upstream LcSVP2 promotes expression of *LcRAP2.4*

**Figure 10:**
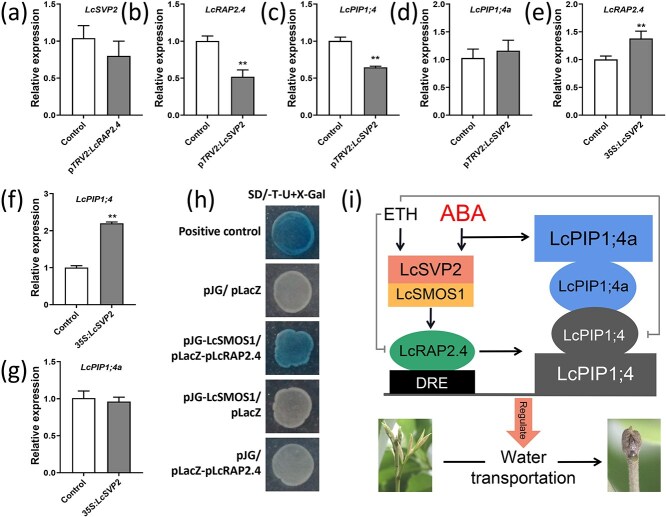
LcSVP2 promotes *LcRAP2.4* expression. (a) Expression levels of *LcSVP2* in p*TRV2:LcRAP2.4* and control buds. (b) Expression levels of *LcRAP2.4*, (c) *LcPIP1;4*, and (d) *LcPIP1;4a* in p*TRV2:LcSVP2* and control buds. (e) Expression levels of *LcRAP2.4*, (f) *LcPIP1;4*, and (g) *LcPIP1;4a* in *35S:LcSVP2* and control buds. (h) The Y1H analysis of the interaction between LcSMOS1 and the *LcRAP2.4* promoter. (i) A model illustrating the role of LcPIPs in regulating the dormancy process of litchi terminal buds. ^**^Indicates a significant difference at *P* < 0.01, as determined by Student’s *t*-test.

Previous studies have demonstrated that LcSVP2 TF can be induced by ETH and ABA, thus promoting the dormancy of litchi terminal buds [4]. Consequently, we speculate that LcSVP2 may also be involved in the LcPIP1;4 regulatory pathway of litchi dormancy. Therefore, *LcSVP2* expression was assessed in the VIGS samples of *LcRAP2.4* (Fig. 10a). The results indicated that there was no significant change in the expression level of *LcSVP2* between the p*TRV2:LcRAP2.4* samples and the control. In contrast, silencing of *LcSVP2* reduced the expression of *LcRAP2.4* and *LcPIP1;4* (Fig. 10b and c), but had no effect on *LcPIP1;4a* expression (Fig. 10d). In addition, transient overexpression of *LcSVP2* in the litchi terminal bud significantly enhanced expression of *LcRAP2.4* and *LcPIP1;4* (Fig. 10e and f), but not *LcPIP1;4a* (Fig. 10g). To investigate their interactive relationship, the Y1H assay was performed to examine the interaction between LcSVP2 and the promoters of *LcRAP2.4* and *LcPIP1;4*. The results demonstrated that LcSVP2 did not interact with the promoters of either *LcRAP2.4* or *LcPIP1;4* (Fig. S7a and b). Furthermore, the Y2H assay indicated that there was no interaction between LcSVP2 and LcRAP2.4 at the protein level (Fig. S7c). These findings suggest that LcSVP2 is positioned upstream of LcRAP2.4 and may indirectly regulate expression of *LcRAP2.4* and *LcPIP1;4*.

Previous studies have also established that LcSVP2 regulates dormancy of litchi terminal buds by interacting with the TF LcSMOS1. Therefore, we hypothesize that LcSVP2 may indirectly influence expression of LcRAP2.4 via LcSMOS1. To test this hypothesis, the Y1H assay was conducted between LcSMOS1 and the *LcRAP2.4* promoter. The results showed that only the positive control and the yeast transformed with both pJG-LcSMOS1 and pLacZ-pLcRAP2.4 turned blue in the SD/−Trp/−Ura medium containing X-gal (Fig. 10h). These findings suggest that LcSMOS1 can directly bind to the promoter of *LcRAP2.4*, while LcSVP2 promotes the expression of *LcRAP2.4* via LcSMOS1.

## Discussion

### The water status of the terminal buds of evergreen litchi varies at different stages

This study first examined changes in water status in the terminal buds of evergreen litchi at various stages. At the dormancy stage, only bound water in the terminal buds was detected by LF-NMR, and the water content was lowest among the four stages. With the breaking of litchi buds, a notable water content was observed, although the proportion of bound water was reduced and that of free water was increased. The highest water content in bud was observed at fast growth stage (S3) with an increased proportion of free water. Prior to reentry of dormancy at S4, the total water content had decreased, while the proportion of free water was diminished and that of bound water had increased. The changes in water status in buds with dormancy onset and bud break in litchi are quite similar to those observed in temperate trees [17–20]. The results indicate that water mobility differs in litchi terminal buds between the dormancy and growth stages. Therefore, further investigations are warranted to clarify the role of PIP AQPs in transmembrane water movement during dormancy onset.

### LcPIP1;4 and LcPIP1;4a are involved in regulation of dormancy onset of litchi terminal buds

Although PIP proteins have been extensively studied in *Arabidopsis* and numerous annual plants, research on these proteins in woody plants, particularly in relation to bud dormancy regulation in evergreen fruit trees, remains scarce. In *Arabidopsis*, the PIP family has experienced functional divergence over time, contributing to the regulation of various developmental processes [41], including plant growth, disease resistance [42–44], and responses to drought and other abiotic stressors [45, 46]. PIPs have also been found to play roles in the regulation of seed dormancy and germination in *Arabidopsis* [47, 48]. In peach trees, *AQPs* have been linked to plant development, which involves cell division and differentiation, as well as significant water utilization, exemplified by the active–dormancy cycle of buds [19, 23]. In the Japanese pear, some water channel-related *AQP* genes are highly expressed during the sprouting of buds, suggesting potential regulatory roles in the dormancy and bud growth cycles of buds [49]. In evergreen tea, the expression of *CsAQP* genes governs flower development and bud endodormancy, and application of dormancy-inducing ABA reduces expression of several *CsAQPs* [50].

A litchi AQP gene *LcPIP1;4*, was found downregulated by dormancy-enforcing ETH in this study. LcPIP1;4 protein was primarily found in the plasma membrane, as other PIP proteins previously reported in *Arabidopsis* [51]. The *Arabidopsis* AQP PIP1;4 was reported to interact with Harpin Hpa1 in the plasma membrane to enhance substrate transport and photosynthesis [52]. Expression of *LcPIP1;4* was detected in all examined tissues, but more prominently expressed in the terminal buds, leaves, flowers, pericarps, and stems, signifying a potential role in the transport of substances associated with the development of these tissues. Since the expression of *LcPIP1;4* was suppressed by exogenous ETH, which enforces bud dormancy [4, 34, 40], *LcPIP1;4* was initially hypothesized to play a negative role in the onset of bud dormancy in litchi. However, the VIGS assay showed that silencing of *LcPIP1;4* delayed dormancy onset, confirming that LcPIP1;4 plays a positive role in the onset of litchi bud dormancy.

Interestingly, LcPIP1;4 was found to interact with the homologous protein LcPIP1;4a, which is also located in the plasma membrane. Both LcPIP1;4 and LcPIP1;4a belong to the PIP1 subclass and exhibit a high degree of amino acid similarity. It has been widely reported that PIP proteins typically form complexes to regulate cellular processes in plants. For example, *Hordeum vulgare* HvPIP2;4, which is localized to the plasma membrane of oocytes, increases water transport activity in oocytes by interacting with HvPIP1;2 [37]. *Selaginella moellendorffii* SmPIP2;1 can form a homotetramer or heterotetramer with SmPIP1;1 or SmPIP2;2, which increases the sensitivity of yeast cells to H_2_O_2_ [35]. In maize, ZmPIP1;1 interacts with ZmPIP1;2, and simultaneous expression of the *ZmPIP1;1* and *ZmPIP1;2* isoforms led to an increase in the osmotic water permeability coefficient [36].

In this study, *LcPIP1;4* and *LcPIP1;4a* were found to be highly expressed in litchi buds during the dormancy stage, indicating that LcPIP1;4 and LcPIP1;4a may play positive roles in inducing bud dormancy. In fact, silencing of either *LcPIP1;4* or *LcPIP1;4a* delayed the onset of litchi bud dormancy, indicating that that these *AQP* genes are involved in onset of dormancy in litchi terminal buds. Furthermore, the simultaneous silencing of *LcPIP1;4* and *LcPIP1;4a* exacerbated the phenotype associated with bud break, further suggesting that these two genes function synergistically in regulating litchi bud dormancy.

### *LcPIP1;4* is activated by transcription factor LcRAP2.4

This study also found that the expression of *LcPIP1;4* is regulated by the upstream TF LcRAP2.4, which is a member of the ERF/AP2 TF family located in the nucleus. ERF/AP2 proteins are known to regulate various physiological processes involved in plant growth [53]. In *Arabidopsis*, the role of RAP2.4 (also known as WIND1) is involved in regulation of cell dedifferentiation [54] and light- and ETH-mediated developmental processes [55]. Additionally, RAP2.4 has potential regulatory functions in shoot regeneration and seed germination [56, 57]. Studies have shown that the AP2/ERF TF PpEBB is involved in bud break of the Japanese pear [58]. In poplar, the AP2/ERF TF EBB3 acts as a positive regulator of bud break, responding to temperature and regulated epigenetically, thereby establishing a direct connection to the activation of the cell cycle during this process [59]. Meanwhile, EBB1 plays a crucial role in regulating the seasonal dormancy of poplar trees [60]. Studies have also suggested that the role of EBB1 in regulating bud break is probably conserved among various woody perennial species, which are significant for both forestry and agriculture [61].

Here, VIGS technology was employed to silence *LcRAP2.4* in the litchi terminal buds, which significantly delayed the onset of bud dormancy. Silencing of *LcRAP2.4* also notably inhibited expression of *LcPIP1;4*. These findings suggest that LcRAP2.4 functions similar to LcPIP1;4 and plays a positive role in the onset of bud dormancy in litchi. Significantly, this study found that LcRAP2.4 had no significant effect on expression of *LcPIP1;4a* and could not bind to the *LcPIP1;4a* promoter. Although promoter analysis indicated the presence of a DRE element in *LcPIP1;4a*, previous studies have demonstrated that the addition of tandem repeat elements can enhance the binding affinity of TFs to promoters [62]. The *cis*-element sequence of the *LcPIP1;4* promoter that interacts with LcRAP2.4 is composed of two tandem repeat DRE elements, which may account for the strong binding affinity. However, the interaction between LcRAP2.4 and the *LcPIP1;4a* promoter may be undetectable due to weak affinity.

### LcSVP2 is an upstream regulatory factor of *LcRAP2.4*, and the expression of *LcPIP1;4*, *LcPIP1;4a,* and *LcRAP2.4* are controlled by ETH and ABA

ABA and ETH induce and maintain dormancy of litchi buds [4, 34, 40]. These two hormones also share the pathway involving *LcSVP2* and *LcSMOS1* in regulating bud dormancy [4]. However, *LcPIP1;4, LcPIP1;4a*, and *LcRAP2.4* responded differentially to exogenous ETH and ABA, as ETH inhibited expression of *LcPIP1;4* and *LcRAP2.4*, whereas ABA promoted expression. Although not significantly influenced by ETH, *LcPIP1;4a* expression was substantially upregulated by ABA. In *Arabidopsis*, studies have reported that PIP protein and ABA coregulate seed germination [47]. ABA in conjunction with PIP2;8 reduces drought tolerance in *Arabidopsis* by preventing the closure of stomata [63]. These findings indicate that the influence of PIP proteins on various physiological developmental processes in plants may also be reliant on ABA activity. RAP2.4 (also known as WIND1 in *Arabidopsis*) mediates ETH-regulated developmental processes [55]. Therefore, the regulatory function of RAP2.4 may be closely related to the availability of ETH.

The VIGS assays confirmed the positive roles of *LcPIP1;4*, *LcPIP1;4a*, and *LcRAP2.4* in bud dormancy onset. The differential or even opposite effects on these genes of ETH and ABA, both of which induce and maintain bud dormancy, suggest that ETH and ABA employ different pathways to regulate bud dormancy in addition to shared pathways, such as the LcVSP2-LcSMOS1 regulatory module reported by Ma *et al.* [4]. ABA-mediated bud dormancy in litchi involves upregulation of *LcPIP1;4*, *LcPIP1;4a*, and *LcRAP2.4*. In addition, this study also revealed that silencing of *LcSVP2* inhibited expression of *LcRAP2.4*, while silencing of *LcRAP2.4* had no significant effect on *LcSVP2* expression, indicating that the key regulatory factor LcSVP2, previously reported to regulate litchi terminal bud dormancy [4], is located upstream of *LcRAP2.4*, *LcPIP1;4*, and *LcPIP1;4a*.

The main function of PIPs is to control water transport [64–67], and bud dormancy onset and release in temperate woody plants is associated with changes in water content, loss of free water with the onset of dormancy, and increased water uptake with dormancy release [17–20, 49]. In evergreen litchi, similar changes in the water status during dormancy onset and release were observed in the terminal bud. It is suggested that the positive role of LcPIP1;4 and LcPIP1;4a in the onset of bud dormancy is mediated by the effects on water transport. The results of the VIGS assays indicate that reduced expression levels of *LcPIP1;4*, *LcPIP1;4a*, and *LcRAP2.4* led to significantly lower moisture content in the terminal bud compared to the control group. This suggests that the high expression of *LcPIP1;4* and *LcPIP1;4a* at the growth cessation stage might accelerate water loss from the terminal buds, causing free water reduction during dormancy onset.

## Conclusion

Taken together, these findings reveal the involvement of PIP AQPs in the regulation of bud dormancy in litchi. Both LcPIP1;4 and LcPIP1;4a play a positive role in bud dormancy onset, and they interact with each other. High expression of *LcPIP1;4* and *LcPIP1;4a* in the buds during the bud growth cessation stage may accelerate the loss of free water associated with bud dormancy onset. *LcPIP1;4* is directly activated by the upstream TF LcRAP2.4, which also plays a positive role in bud dormancy onset. *LcPIP1;4*, *LcPIP1;4a*, and *LcRAP2.4* are regulated by ETH and ABA, and *LcRAP2.4* is positioned downstream of LcSVP2. Based on these findings and a previous report by Ma *et al.* [4], we propose a model illustrating the synergistic regulation of bud dormancy in litchi by LcPIPs (Fig. 10i). LcRAP2.4 directly binds to the promoter of *LcPIP1;4* to activate its transcription, whereas LcPIP1;4 interacts with LcPIP1;4a, and both can form complexes with themselves. Meanwhile, ABA promotes expression of *LcRAP2.4*, *LcPIP1;4*, and *LcPIP1;4*a, whereas ETH inhibits their expression. In the presence of both ETH and ABA, the promoting effect of ABA on *LcRAP2.4*, *LcPIP1;4*, and *LcPIP1;4*a is predominant. Additionally, LcSVP2, induced by ETH and ABA, acts upstream of *LcRAP2.4*, enhancing its expression, ultimately increasing formation of the LcPIP1;4-LcPIP1;4a complex, which accelerates water loss and promotes the dormancy of litchi terminal buds.

## Materials and methods

### Plant materials

This study was conducted at the experimental orchard in South China Agricultural University, and at the demonstration orchard in Conghua Litchi Expo Park, Guangzhou, China. Trees of litchi cv. ‘Feizixiao’ (16 to 18 years old) were used as the materials. Terminal buds were collected at four developmental stages: dormant (Stage I, S1), bud break (Stage II, S2), rapid growth (Stage III, S3), and growth cessation (Stage IV, S4), following the methodology established by Zhang *et al.* [7]. Additionally, tender roots, mature stems and leaves, and terminal buds were collected from 16-year-old litchi trees on 12 July 2022; fully blooming flowers and mature fruit were gathered in March and June 2022, respectively. The flesh and pericarp were separated from the intact fruit. Tobacco (*Nicotiana benthamiana*) plants were transplanted and grown in a culture room maintained at 23°C for approximately 20 days under a 16-h light cycle.

### Water status analyses in litchi terminal buds

The water spectrum in the litchi shoot buds was analyzed using an LF-NMR system (NM12-060H-I; Niumag Analytical Instrument, Jiangsu). This system allows for the assessment of hydrogen proton relaxation, which is essential to assess water mobility and material distribution [68]. The transverse relaxation time (T2) for each sample’s spectrum was recorded utilizing the pulse Carr-Purcell-Meiboom-Gill (CPMG) sequences, following the methods reported in an earlier study [69]. The settings of the CPMG sequences were a dominant frequency (SF) of 12 MHz; an RF delay of 0.02 ms; 90° (P1) and 180° (P2) radio-frequency pulse widths of 7.52 and 14.48 μs, respectively; a waiting time for repeated sampling (TW) of 1200 ms; a signal sampling points (TD) of 1024; a digital gain during prescanning (DRG) and a preamplification gain during prescanning (PRG) of 3 dB; and an analog gain during prescanning (RG) of 20 dB. Additionally, the samples were examined using a multilayer spin echo pulse sequence of LF-NMR, resulting in images depicting the moisture content of litchi buds. MRI was performed to assess the internal distribution of moisture in the buds during the different stages [70]. For MRI analysis, TW was set at 1000 ms, RG at 20 dB, PRG at 3 dB, DRG at 5 dB, and echo time at 4.21 ms, with an average number of 16. Data were analyzed using Niumag Multi-Exp Inv Analysis software. T2 is highly correlated with water mobility. Water phase at T2 ranging from 0 to 10 ms (T21) is considered bound water, which is almost immobile; that ranging from 10 to 100 ms (T22) is semibound water with higher fluidity, and that ranging from 100 to 1000 ms (T23) is classified as highly mobile free water.

The moisture content was assessed using the wet basis method. The determination of water content in the bud samples followed the guidelines outlined in the *Detection of Water Content in Foods* [71]. Samples weighing between 2 and 5 g were placed in a drying oven set at 105°C for a duration of 2 to 4 h. Once taken out of the oven, the samples were sealed and allowed to cool in a desiccator for 30 min prior to weighing. They were then returned to the oven at 105°C for an additional hour, followed by sealing and cooling in a desiccator for another 30 min prior to weighing. The process was carried out repeatedly until the difference in mass fell below 2 mg; at that point, the weight was logged as the mass of the dry matter. The water content was then determined using a formula established in a prior study [69].

### Gene amplification and vector construction

To obtain the coding sequences (CDSs) of *LcPIPs* and *LcRAP2.4*, PCR was performed with the degenerate primers based on *LcPIPs* and *LcRAP2.4* sequences, which was obtained from the http://www.sapindaceae.com/ database. Gene-specific primers listed in Table S3 for PCR were designed using Primer 5. The PCR amplification program consisted of an initial denaturation step at 95°C for 2 min, followed by 36 cycles of denaturation at 95°C for 20 s, annealing at 55°C for 20 s, and extension at 72°C for 45 s, and a final extension step at 72°C for 10 min. The final PCR products were purified and cloned into the pMD18-T plasmid for sequencing.

### RNA extraction and qRT-PCR assays

Total RNA was extracted from litchi using the TRIzol-RNA Extract kit provided by Thermo Fisher Scientific. The first-strand cDNA was synthesized from a reaction mixture comprising 1 μg of RNA, 2 μl of PrimeScript buffer, and a minimum of 10 μl of RNase-free H_2_O, employing the PrimeScript kit (TaKaRa, Japan). qRT-PCR was conducted on an Applied Biosystems Quan StudioTM 7 Flex Real-Time PCR System (Thermo Fisher, CA, USA), following a previously established method [72]. ITaq™ Universal SYBR Green Supermix was employed in these assays. The thermal cycling parameters were set at 37°C for 45 min, followed by 85°C for 5 s, and concluded at 4°C for 5 min. The qRT-PCR procedure began with an initial denaturation step at 94°C for 6 min, which was succeeded by 45 cycles of denaturation at 94°C for 20 s and annealing at 60°C for 30 s. The reaction mixture, totaling 10 μl, included 100 ng of cDNA, 0.2 μmol/ml of an appropriate gene-specific primer, and 6 μl of PCR mix. Each analysis included three biological replicates of trees, each with three technical replicates. The relative expression levels were determined using the 2^−△△CT^ method, with litchi *LcActin* serving as an internal reference gene. The primers utilized in this research are detailed in Table S3.

### Phylogenetic analysis and sequence analysis

A phylogenetic tree that included PIP or RAP proteins was constructed using the MEGA 7 software with the neighbor-joining (NJ) method [73]. The bootstrap parameter was established with 1000 bootstrap replicates. Visualization of the phylogenetic tree was accomplished through iTOL (https://itol.embl.de/). Alignment of the PIPs or RAPs protein sequences was conducted with the ClustalX [74]. The promoter sequences of *LcPIPs* were analyzed with the PlantCARE database.

### Transcriptional activation activity analysis of LcRAP2.4

The complete CDS of *LcRAP2.4* was ligated to the pGBKT7 (BD) vector to create the BD-LcRAP2.4 fusion expression vector. The primers listed in Table S3 for full-length amplification were designed based on the polyclonal sites and the *LcRAP2.4* sequence present in the vector, which included EcoRI and BamHI restriction sites. The pGBKT7-53 and pGADT7-T plasmids combination functioned as a positive control, whereas the empty pGBKT7 vector was utilized as a negative control. The constructed vector was successfully sequenced and then transformed into AH109 yeast cells. Positive detection was carried out on the selected monoclonal strains. Subsequently, the positive yeast cells along with the control yeast were inoculated onto SD/−Trp and SD/−Trp/−His/−Ade + X-α-gal media, then incubated in the dark at 30°C for 3 days to evaluate yeast cell growth. The primers utilized are detailed in Table S3.

### Subcellular localization assays of LcPIP1;4/a and LcRAP2.4

The open reading frames of *LcPIP1;4/a* and *LcRAP2.4* were amplified using PCR and subsequently cloned into the pBI121-GFP vector, resulting in the creation of the recombinant expression vectors pBI121-LcPIP1;4/a-GFP and pBI121-LcRAP2.4-GFP. These vectors were then introduced into the GV3101 *Agrobacterium tumefaciens* through the freeze–thaw technique method [75]. For transient transformation, *N. benthamiana* leaves were infiltrated with a suspension of *Agrobacterium* cells. After a 3-day incubation period, GFP fluorescence was detected using a confocal laser-scanning microscope (LSM800, Zeiss Microscopy, Jena, Germany). The primers utilized are detailed in Table S3.

### Yeast assays

In the yeast one-hybrid (Y1H) assay, the CDS of *LcRAP2.4* was amplified via PCR from litchi and subsequently cloned into the pGADT7 vector (Clontech, USA) to create the pGADT7-LcRAP2.4 construct. The *LcPIP1;4* promoter fragment was also amplified and inserted into the pAbAi vector to create the pAbAi-LcPIP1;4 construct. For the transformation process of yeast cells, the Matchmaker Gold Y1H system (Clontech, Takara) was utilized following the manufacturer’s instructions. Briefly, the linearized pAbAi vector, which includes the promoter fragment of *LcPIP1;4*, was integrated into the genome of the Y1H Gold yeast strain. The AD-LcRAP2.4 vector and the empty pGADT7 vector were introduced into the Gold yeast strain. As a positive control, the yeast Y1H Gold was cotransformed with pGADT7-53 (AD-53) and the p53-promoter. Positive yeast cells were isolated on SD/−Leu plates containing 500 ng/ml of aureobasidin A (AbA) and incubated at 30°C for a duration of 3 days to assess the interaction between the LcRAP2.4 protein and the *LcPIP1;4* promoter. The Y1H assays conducted to examine the interactions between LcRAP2.4 and the *LcPIP1;4a* promoter, as well as LcSVP2 and the promoters of *LcRAP2.4* and *LcPIP1;4*, and LcSMOS1 and the *LcRAP2.4* promoter, were performed according to the methodology described in a previous study [76].

The CDS of LcPIP1;4a was utilized as the bait in the yeast two-hybrid (Y2H) assay, following the Dual Systems Biotech protocol [77]. The bait construct was generated by PCR amplification of LcPIP1;4a cDNA and was cloned into the pBT3-STE vector via the *Sfil* site, resulting in the pBT3-STE-LcPIP1;4a construct, which encodes a fusion protein with VP16-LexA-Cub attached to the C-terminus of LcPIP1;4a. The bait constructs were introduced into the NMY51 strain using standard transformation techniques [78]. The lack of self-activation was confirmed by cotransforming the bait with the control prey pNubG-Fe65 and selecting on a medium devoid of leucine, tryptophan, histidine, and adenine. Additionally, *LcPIP1;4* was amplified and subcloned into the pPR3-N vector to form the pPR3-N-LcPIP1;4 construct. The pBT3-STE-LcPIP1;4a and pPR3-N-LcPIP1;4 constructs were then transformed into NMY51; pTSU2-APP and pNubG-Fe65 served as positive controls, while pPR3-N and pTSU2-APP acted as negative controls. The Y2H system used to investigate the interaction between the TFs LcSVP2 and LcRAP2.4 follows the methodology described in a previous study [4]. The primers utilized are detailed in Table S3.

### BiFC assay

The *LcPIP1;4* and *LcPIP1;4a* CDSs were cloned into the pSPYNE (LcPIP1;4-YFP^N^) and pSPYCE (LcPIP1;4a-YFP^c^) vectors, respectively, and individually introduced into *Agrobacterium* strain GV3101. Equal volumes and concentrations of the LcPIP1;4-YFP^N^ and LcPIP1;4a-YFP^c^  *Agrobacterium* strains were combined and subsequently injected into the leaves of 3-week-old *N. benthamiana*. Fluorescence signals were detected after 3 days of infiltration using a confocal laser scanning microscope (LSM800, Zeiss, Germany). The primers are detailed in Table S3.

### Electrophoretic mobility shift assays

The open reading frame of *LcRAP2.4* was inserted into the pGEX-4 T vector to generate the fusion construct pGEX-4 T-LcRAP2.4, which was subsequently transformed into competent *Escherichia coli* BL21 cells. Expression of the recombinant protein was triggered by the addition of isopropyl β-d-1-thiogalactopyranoside, and EMSAs were conducted utilizing a LightShift™ Chemiluminescent EMSA Kit (Thermo Fisher Scientific) following the provided guidelines. Initially, a nondenaturing polyacrylamide gel was prepared, and the assay involved steps of spotting, electrophoresis, film transfer, washing, and development.

### Dual-luciferase and LCI assays

The CDS of LcRAP2.4 and the promoter sequence of *LcPIP1;4* were inserted into the pGreen II 62-SK and pGreen II 0800-LUC vectors, respectively, which were then subjected to double digestion with the BamHI and SalI restriction enzymes. For the experimental group, *Agrobacterium* cultures containing the effector and reporter vectors at a 9:1 ratio were introduced into the leaf cells of 3-week-old *N. benthamiana*. The control group received only the reporter vector. After injection, the samples were incubated in the dark at room temperature for 3 days. Afterward, the activities of the firefly and *Renilla* luciferases were assessed using a Dual Luciferase Reporter Gene Assay Kit (YEASEN, China). Measurements of three biological replicates are expressed as the LUC/REN ratio. The primers utilized are detailed in Table S3.

For the LCI assays, the CDSs of LcPIP1;4 and LcPIP1;4a were linked to the N-terminal luciferase (pCAMBIA1300-nLUC) and the C-terminal luciferase (pCAMBIA1300-cLUC), respectively. The primers used for constructing the vectors are detailed in Table S3. *Agrobacterium* strain GV3101, containing the respective constructs, was coinfiltrated into the leaves of *N. benthamiana* through *A. tumefaciens*-mediated coinfiltration. After allowing the plants to incubate at 25°C for 3 days, 1 mmol/l of d-luciferin sodium salt (Coolaber, China) was injected at the abaxial surface of the leaf, following the protocol outlined in a previous study [79]. The luminescence of luciferase (LUC) was then measured using the Night Shade LB 985 system (Berthold, Bad Wildbad, Germany).

### VIGS assays

Gene silencing in the litchi terminal buds was carried out following an established method using a VIGS system [4]. A vector based on the tobacco rattle virus (TRV) was employed in this experiment. Specifically, approximately 500-bp fragments of the CDSs of *LcPIP1;4*, *LcPIP1;4a*, and *LcRAP2.4* were cloned into the TRV2 vector to create the constructs pTRV2-LcPIP1;4, pTRV2-LcPIP1;4a, and pTRV2-LcRAP2.4, respectively. The pTRV1 and the fusion vector plasmids were individually introduced into the *A. tumefaciens* strain GV3101. The *Agrobacterium* cells containing the pTRV1 and the fusion vectors were combined with a buffer solution of 10 mM 2-(N-morpholino) ethanesulfonic acid, 10 mM MgCl_2_, and 200 mM acetosyringone at pH 5.6. The infection solution was prepared by mixing pTRV1 and pTRV2 or the fusion vectors in a 1:1 volume ratio, with the combination of pTRV1 and pTRV2 serving as a negative control. Transformation of litchi buds at the S3 stage was carried out through stem injection at 6 mm beneath the terminal bud. After approximately 21 days, the buds were analyzed using qRT-PCR, and the VIGS group exhibiting reduced expression of target genes was selected for further investigation. Bud morphology was documented through photographs, and the number of breaking terminal buds was recorded. The primers utilized are detailed in Table S3.

### Hormone treatments

Three 18-year-old adult ‘Feizixiao’ litchi trees were chosen for hormone treatment. The terminal buds at stage 1 (S1) served as the subjects for this experiment, and they were classified into four groups: control, ETH, ABA, and ABA + ETH. For each treatment, the terminal buds were split into two equal sections, which were randomly allocated to receive either a spray of clean water (control) or the respective hormones. The control group was treated with water mixed with 0.05% Tween-20. The concentrations of ABA and ETH treatments were 200 and 1000 mg/l, both containing 0.05% Tween-20, respectively. Bud samples were collected at 0, 12, 24, and 72 h post-treatment. The number of broken buds was recorded at 0, 30, and 45 days, and the germination rate was subsequently calculated.

### Statistical analysis

Date analysis and visualization were conducted using GraphPad Prism V8.0 (GraphPad Software, LLC, San Diego, CA, USA). The results are presented as the means ± standard deviations. Comparisons were made using the two-tailed *t*-test or Duncan’s multiple range test in conjunction with ANOVA. A probability (*P*) value <0.05 was considered statistically significant. The qRT-PCR assays were performed in triplicate (*n* = 3) to evaluate repeatability.

## Supplementary Material

Web_Material_uhaf122

## Data Availability

All the data used in this research can be found within the main text or the supplementary materials, and interested parties can request the raw data from the corresponding author with a reasonable justification.
