# Supplementary figures and images for: Two aquaporins, LcPIP1;4 and LcPIP1;4a, cooperatively regulate the onset of dormancy of the terminal buds in evergreen perennial litchi (Litchi chinensis Sonn.)
Source: Hortic Res. 2025 May 7;12(8):uhaf122. doi: 10.1093/hr/uhaf122 (PMC12261106; doi:10.1093/hr/uhaf122)

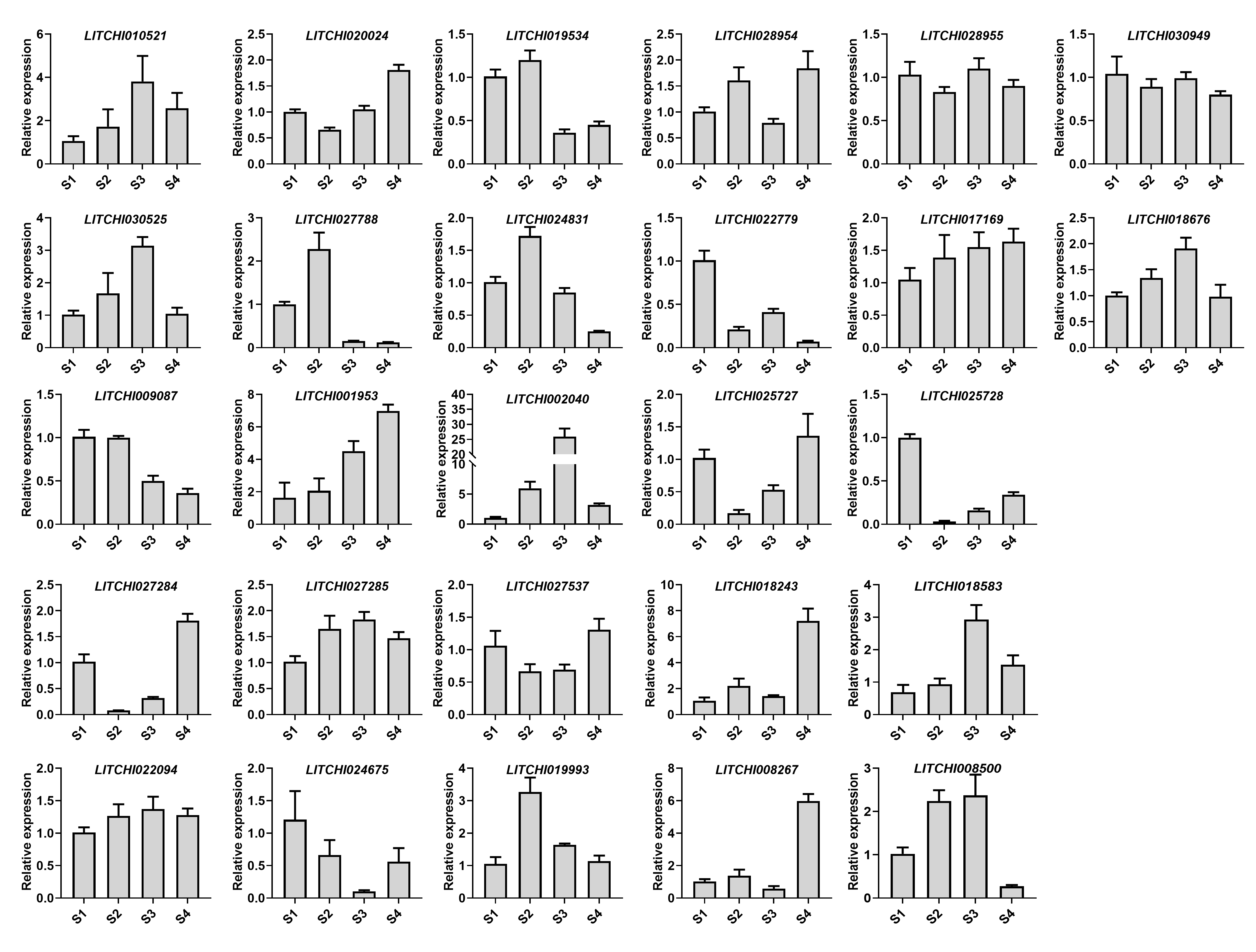

Supplement: Web_Material_uhaf122 [file web_material_uhaf122.zip › Figure S1.jpg]

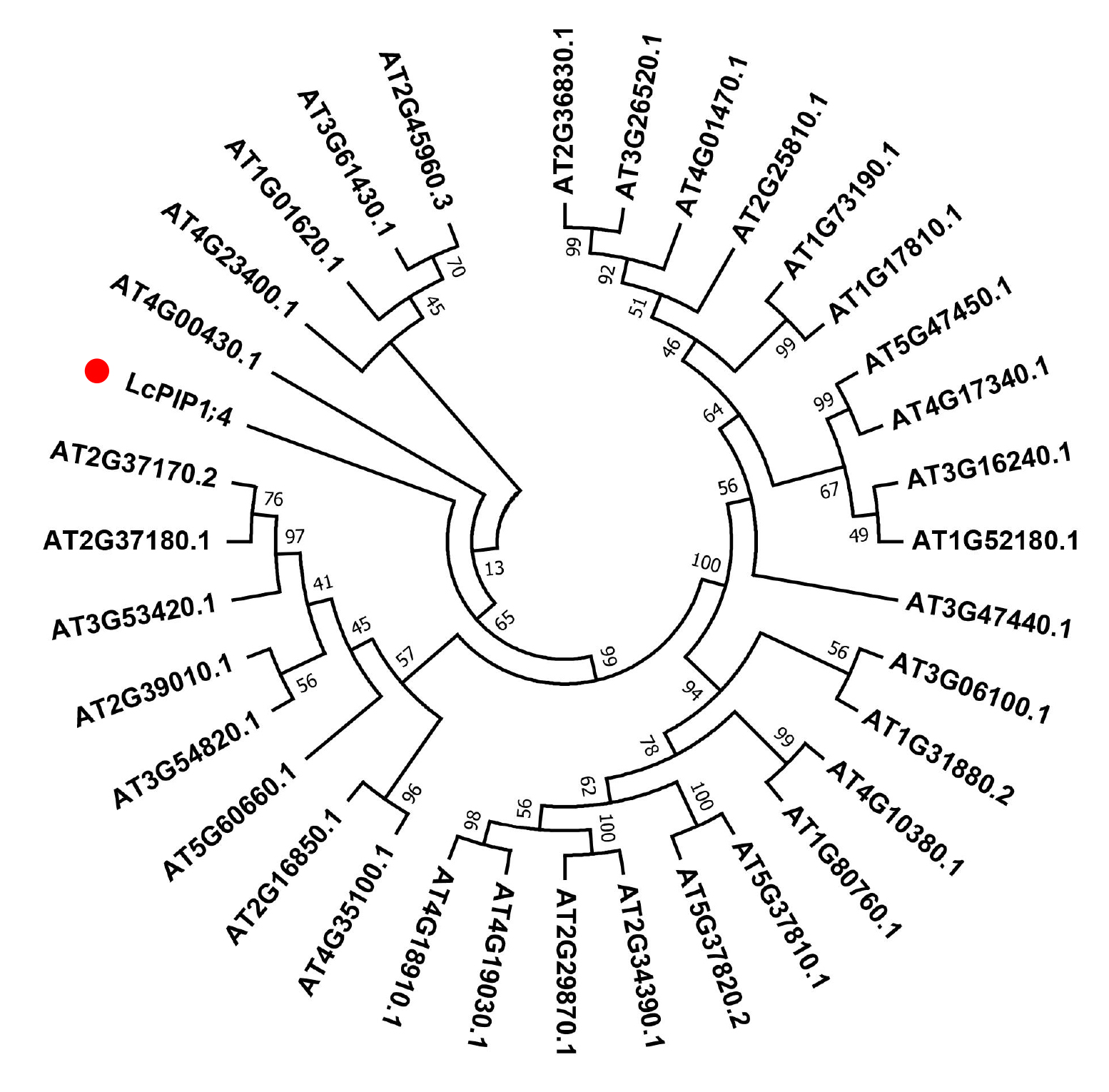

Supplement: Web_Material_uhaf122 [file web_material_uhaf122.zip › Figure S2.jpg]

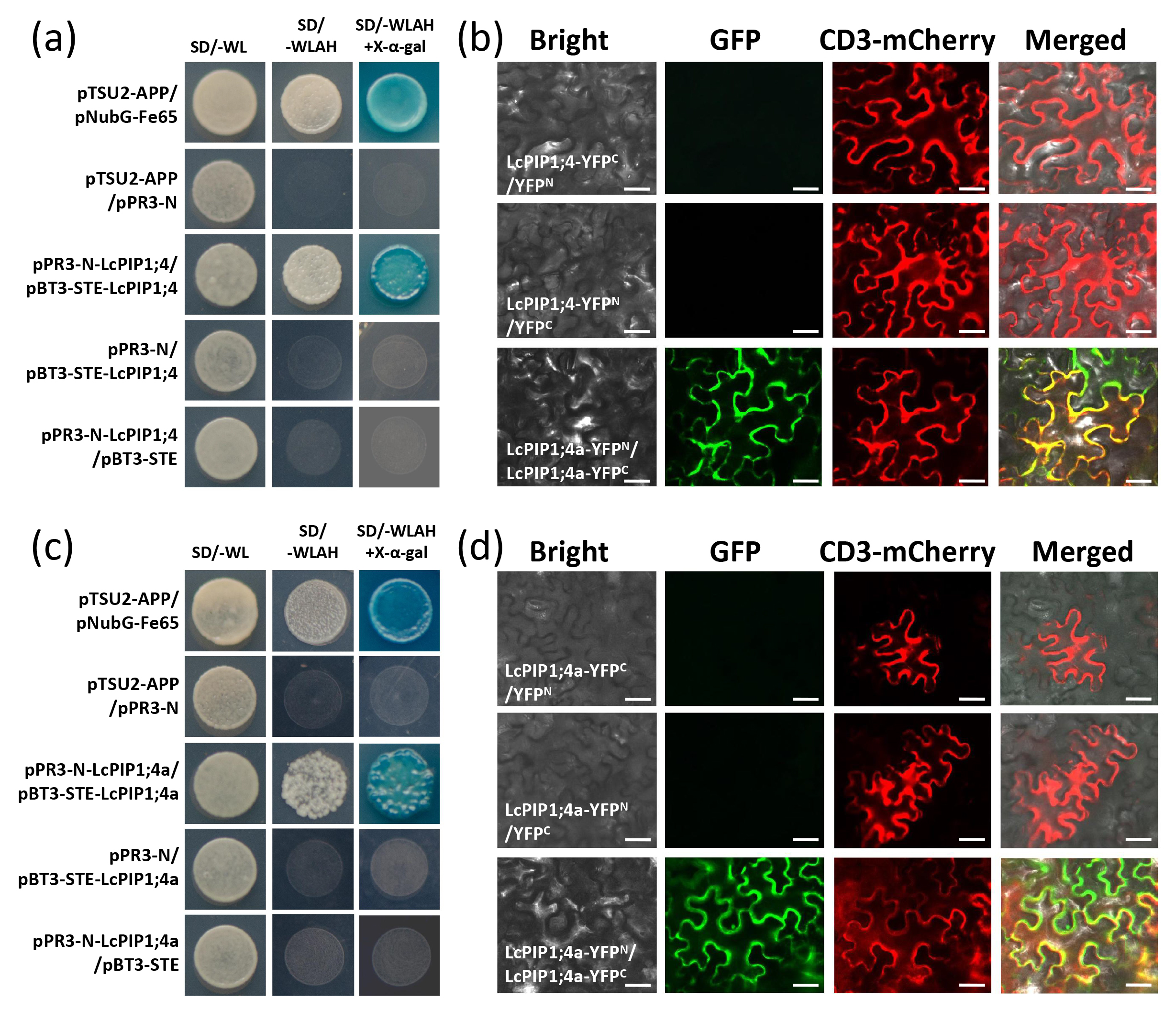

Supplement: Web_Material_uhaf122 [file web_material_uhaf122.zip › Figure S3.jpg]

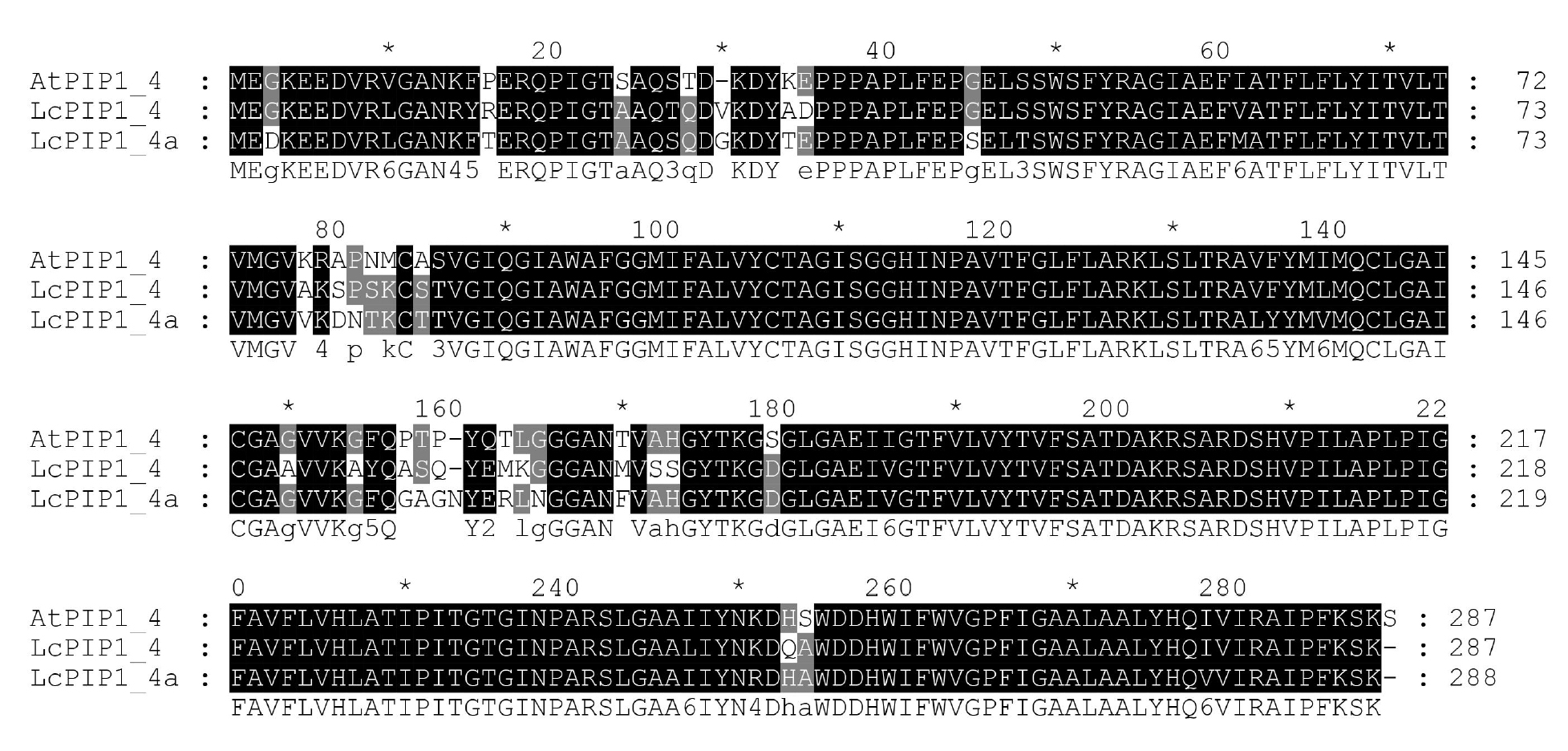

Supplement: Web_Material_uhaf122 [file web_material_uhaf122.zip › Figure S4.jpg]

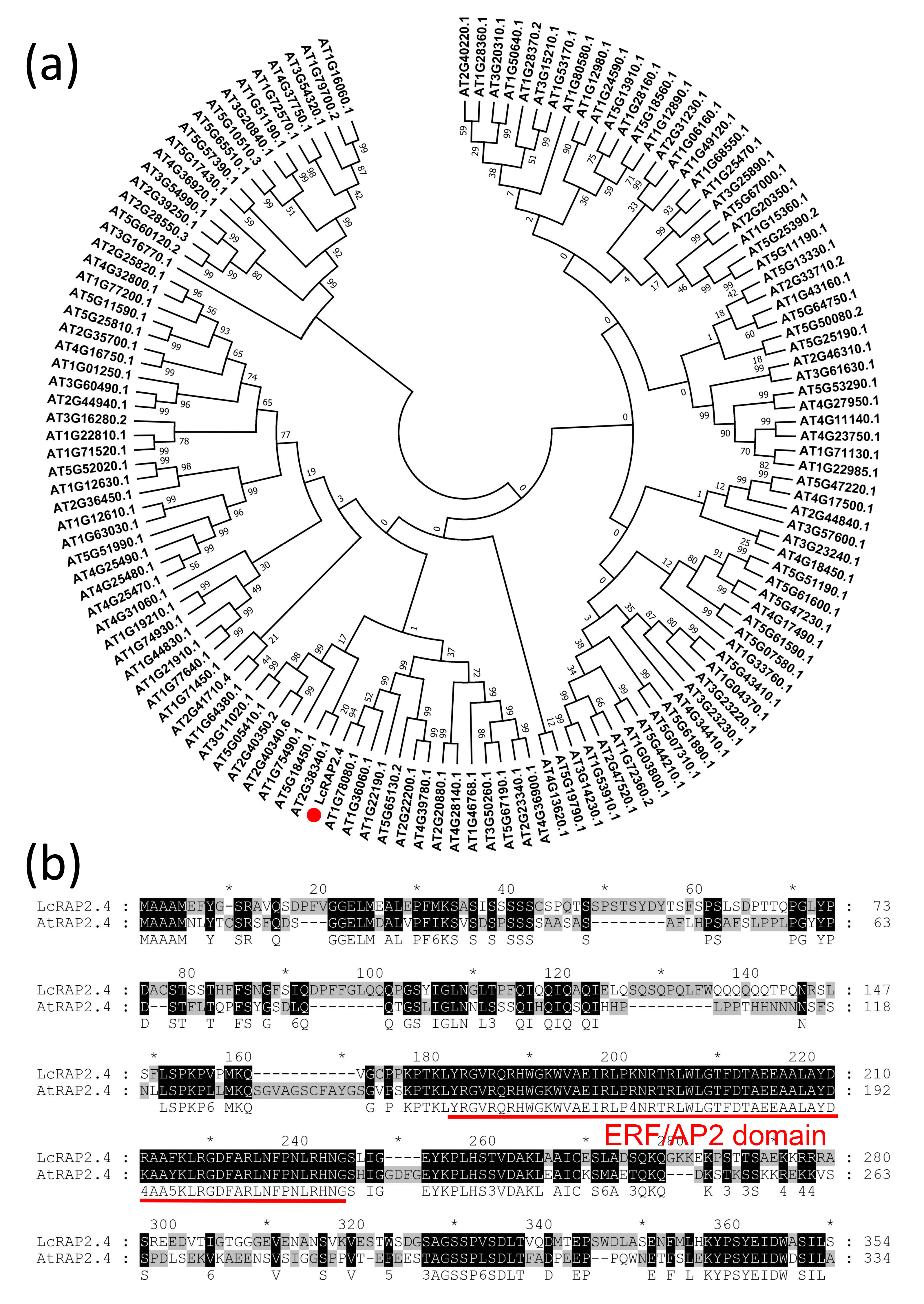

Supplement: Web_Material_uhaf122 [file web_material_uhaf122.zip › Figure S5.jpg]

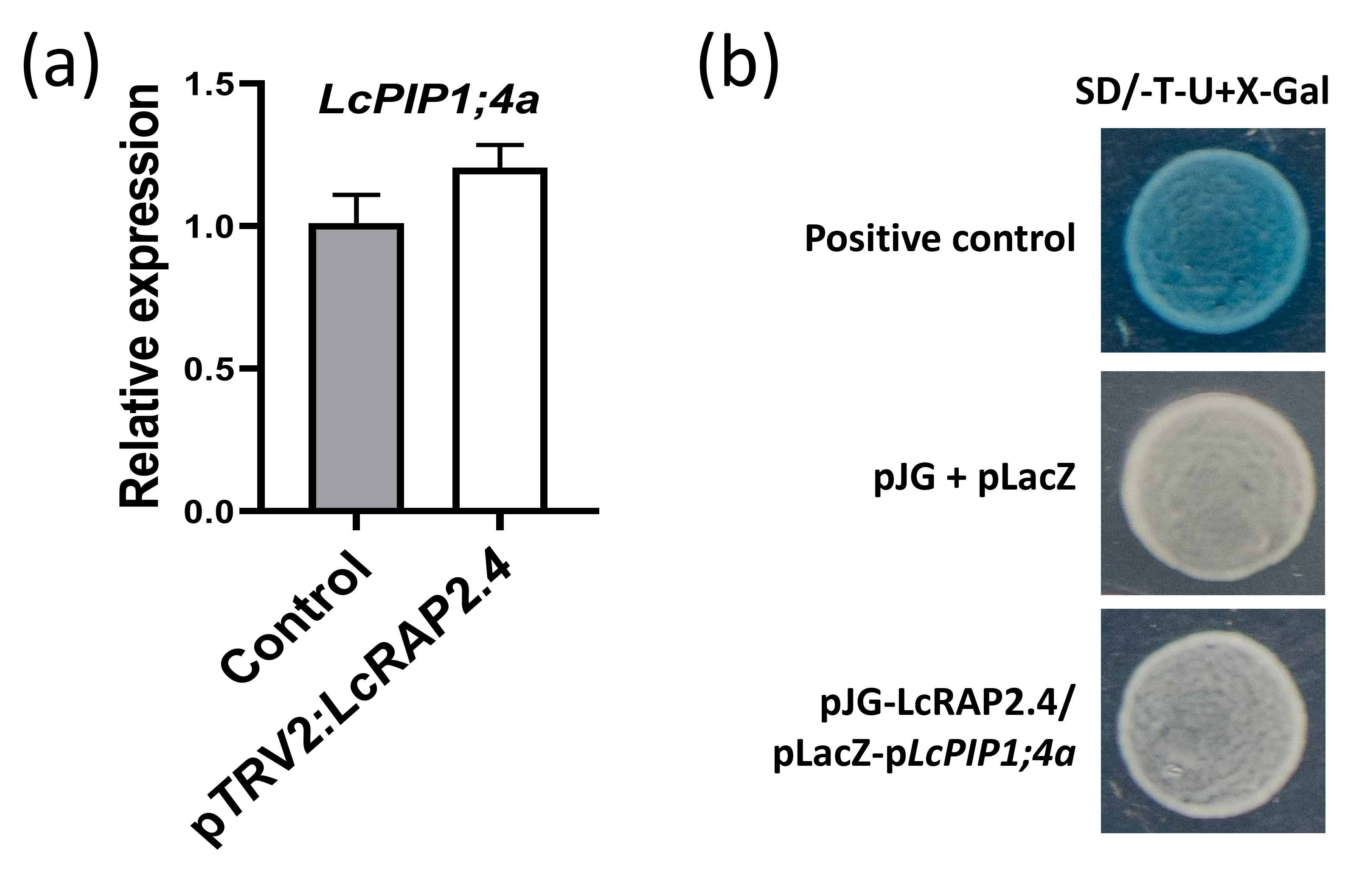

Supplement: Web_Material_uhaf122 [file web_material_uhaf122.zip › Figure S6.jpg]

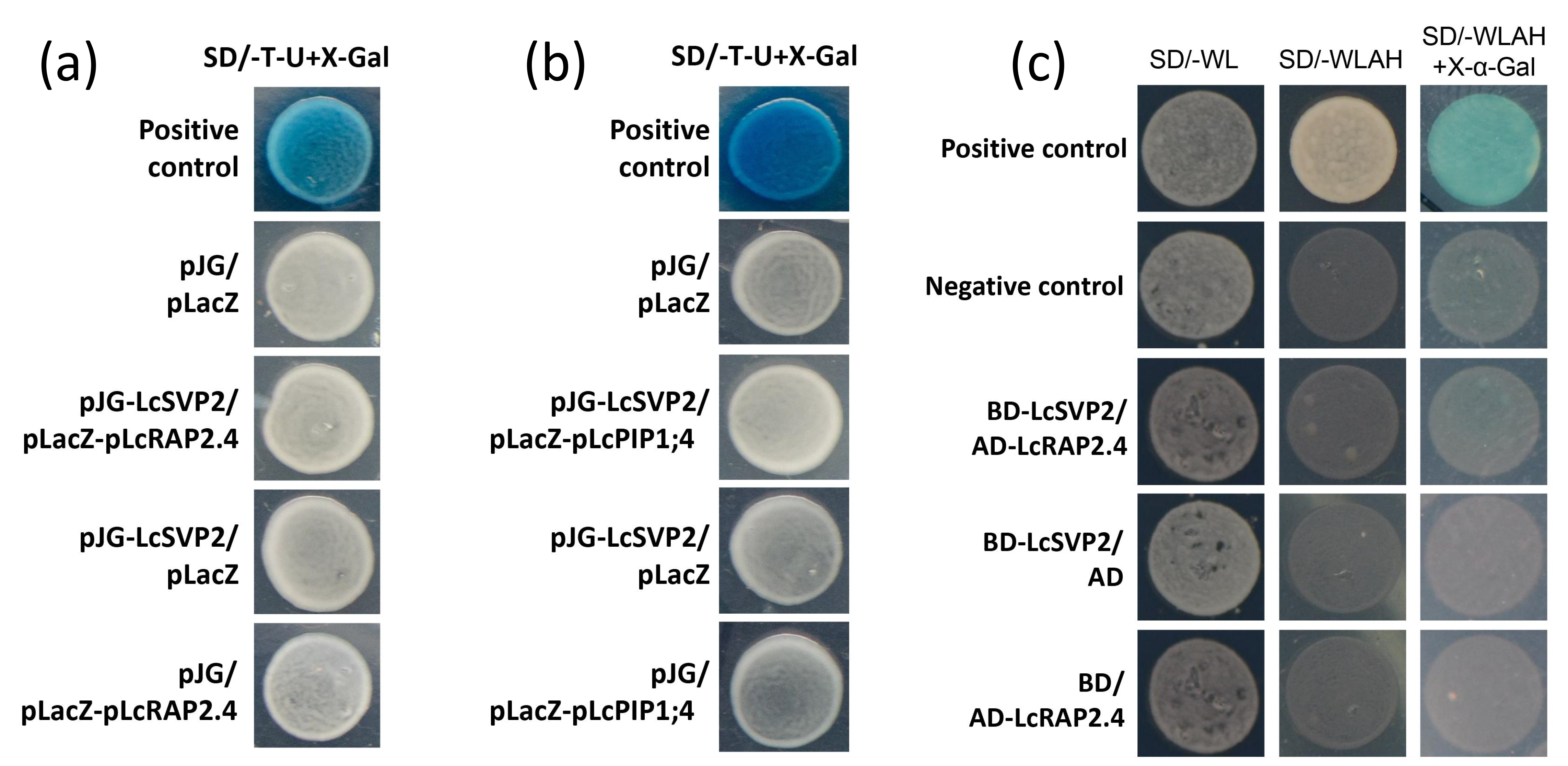

Supplement: Web_Material_uhaf122 [file web_material_uhaf122.zip › Figure S7.jpg]
